# Kinetics and specificity of paternal mitochondrial elimination in *Caenorhabditis elegans*

**DOI:** 10.1038/ncomms12569

**Published:** 2016-09-01

**Authors:** Yang Wang, Yi Zhang, Lianwan Chen, Qian Liang, Xiao-Ming Yin, Long Miao, Byung-Ho Kang, Ding Xue

**Affiliations:** 1 School of Life Sciences and Collaborative Innovation Center for Diagnosis and Treatment of Infectious Diseases, Tsinghua University, Beijing 100084, China; 2 Institute of Biophysics, Chinese Academic of Sciences, Datun Road, Chaoyang District, Beijing 100101, China; 3 Tsinghua University-Peking University Joint Center for Life Science, Beijing 100084, China; 4 Departments of Pathology and Laboratory Medicine, Indiana University School of Medicine, Indianapolis, Indiana USA; 5 State Key Laboratory of Agrobiotechnology, Cellular and Molecular Biology Program, School of Life Sciences, The Chinese University of Hong Kong, New Territories, Hong Kong, China; 6 Department of Molecular, Cellular, and Developmental Biology, University of Colorado, Boulder, Colorado 80309, USA

## Abstract

In most eukaryotes, mitochondria are inherited maternally. The autophagy process is critical for paternal mitochondrial elimination (PME) in *Caenorhabditis elegans*, but how paternal mitochondria, but not maternal mitochondria, are selectively targeted for degradation is poorly understood. Here we report that mitochondrial dynamics have a profound effect on PME. A defect in fission of paternal mitochondria delays PME, whereas a defect in fusion of paternal mitochondria accelerates PME. Surprisingly, a defect in maternal mitochondrial fusion delays PME, which is reversed by a fission defect in maternal mitochondria or by increasing maternal mitochondrial membrane potential using oligomycin. Electron microscopy and tomography analyses reveal that a proportion of maternal mitochondria are compromised when they fail to fuse normally, leading to their competition for the autophagy machinery with damaged paternal mitochondria and delayed PME. Our study indicates that mitochondrial dynamics play a critical role in regulating both the kinetics and the specificity of PME.

## Abstract (web summary)

Autophagy mediates the degradation of paternal mitochondria after fertilization in *C. elegans* to ensure that mitochondria are inherited maternally. Here the authors show that mitochondrial dynamics is critical for the selectivity and kinetics of paternal mitochondrial elimination.

## Graphical abstract

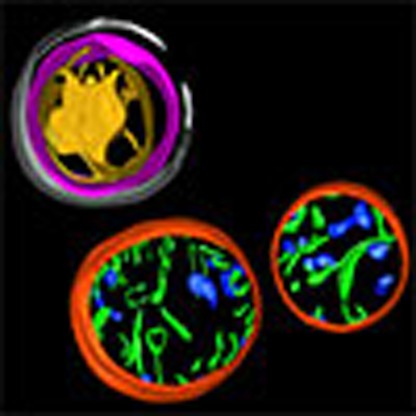

Mitochondria are versatile organelles that mediate multiple essential cellular functions, including energy production, apoptosis and stress responses^1^,^2^. They carry their own genomes (mtDNA), which encode multiple subunits of the oxidative phosphorylation complexes as well as transfer and ribosomal RNAs (tRNAs and rRNAs)^2^,^3^,^4^. In most animals, mitochondria are maternally inherited, and paternal mitochondria are selectively eliminated from developing embryos^5^,^6^,^7^,^8^. Recent studies reveal that this phenomenon is conserved in *Caenorhabditis elegans*, where paternal mitochondria and mtDNA are selectively eliminated during early embryogenesis^9^,^10^,^11^. Autophagy, a major cellular degradation process that proceeds through formation of autophagosomes with double-layer membranes enclosing damaged or unnecessary organelles^12^,^13^, is partially responsible for paternal mitochondrial elimination (PME), leading to their eventual degradation through lysosomes. However, it has been enigmatic how the autophagy machinery in the embryo can selectively target paternal mitochondria, but not maternal mitochondria, for degradation.

Autophagy of mitochondria plays a critical role in normal cells, acting to control mitochondrial quality by eliminating old and damaged mitochondria^14^,^15^,^16^,^17^,^18^. Interestingly, this autophagy process, also called mitophagy, is closely associated with the mitochondrial fusion and fission processes, which generate tubular, elongated mitochondria and fragmented mitochondria, respectively, and are coordinated in normal cells to control appropriate mitochondrial morphology and dynamics^19^,^20^. Reduced mitochondrial fission or increased mitochondrial fusion can protect against mitophagy^16^,^21^,^22^,^23^. For example, during nutrient starvation, normal cells with elongated mitochondria are protected from autophagy elimination, while fusion-deficient cells, in which mitochondria become fragmented, do not survive^22^,^23^. These observations suggest that mitochondrial dynamics play an important role in regulating appropriate mitophagy and the functions and survival of cells.

In this study, we demonstrate that the mitochondrial fission and fusion processes in both sperm and oocytes play an important role in regulating the kinetics and specificity of PME. A defect in paternal mitochondrial fission and a defect in maternal mitochondrial fusion synergize to greatly delay PME, whereas a defect in paternal mitochondrial fusion accelerates PME. Importantly, we find that paternal mitochondria are permeabilized and depolarized upon entry into the oocyte and are rapidly surrounded by autophagosomes. This rapid and highly specific targeting of paternal mitochondria by autophagosomes is crippled when maternal mitochondria are compromised due to a defect in mitochondrial fusion and compete with damaged paternal mitochondria for the autophagy machinery. Our study thus provides critical insights into the long-standing, fundamental question of how paternal mitochondria are specifically distinguished from maternal mitochondria during the PME process.

## Results

### Mitochondrial dynamics in worm sperm and embryos

We investigated if mitochondrial dynamics play a role in maternal inheritance of mitochondria. *C. elegans fzo-1* and *drp-1* genes are required for fusion and fission of mitochondria, respectively^24^,^25^,^26^,^27^,^28^, and encode GTPases of the dynamin family that are orthologues of the MFN1/FZO1 protein, which mediates mitochondrial fusion, and the DRP1 protein, which promotes mitochondrial fission^29^,^30^,^31^,^32^,^33^. Two strong loss-of-function (*lf*) mutations, *tm1108* and *tm1133*, eliminate the activity of *drp-1* and *fzo-1* and cause severe mitochondrial fission and fusion defects, respectively^26^. For example, *C. elegans* embryos stained with tetramethylrhodamine ethyl ester (TMRE), a mitochondria-specific dye, showed distinct morphology and connectivity of the mitochondrial network in different mutant backgrounds^25^,^26^ (Supplementary Fig. 1). In wild-type (N2) embryos, mitochondria display different shapes and sizes and are broadly distributed in the cytoplasm of each blastomere (Supplementary Fig. 1a). In *drp-1(tm1108)* embryos that are defective in mitochondrial fission, long and highly connected mitochondria are observed, which often exist as asymmetric, concentrated clusters in the blastomeres (Supplementary Fig. 1b). In *fzo-1(tm1133)* embryos that are defective in mitochondrial fusion, mitochondria are highly fragmented and stained poorly by TMRE, with no obvious mitochondrial network observed (Supplementary Fig. 1c,d). Interestingly, in *fzo-1(tm1133); drp-1(tm1108)* double mutant embryos, long and highly connected mitochondria are observed, which are indistinguishable from those seen in *drp-1(tm1108)* embryos (Supplementary Fig. 1b,e), indicating that the mitochondrial fusion defect caused by *fzo-1(tm1133)* is completely suppressed by the mitochondrial fission defect mediated by *drp-1(tm1108).*

Electron microscopy (EM) analysis of *C. elegans* embryos is consistent with the observations from the fluorescence microscopy analysis. In wild-type *C. elegans* embryos, mitochondria display a variety of shapes and sizes, ranging from small spherical organelles to longer dumbbell-shaped organelles^25^,^26^,^27^. In *drp-1(tm1108)* embryos, mitochondria fail to fission and are often very long, resulting in fewer individual mitochondria in each cell. Mitochondria are highly fragmented and spherical in *fzo-1(tm1133)* embryos, in which mitochondrial fusion fails to occur and fission proceeds^25^,^26^. We performed EM analysis of spermatozoa from N2, *drp-1(tm1108)* and *fzo-1(tm1133)* males and observed somewhat different mitochondrial morphology. Mitochondria are mostly spherical in N2 sperm, with an average diameter around 0.5 μm (Supplementary Fig. 2a,d). Larger and longer mitochondria are often observed in *drp-1(tm1108)* sperm, but they are fewer in number (Supplementary Fig. 2b,d). Mitochondria are spherical in *fzo-1(tm1133)* sperm (Supplementary Fig. 2c); however, unlike in *fzo-1(tm1133)* embryos, where mitochondria are significantly smaller than those in N2 embryos^25^,^26^, mitochondria in *fzo-1(tm1133)* sperm are slightly larger in diameter and fewer in number than mitochondria in N2 sperm (Supplementary Fig. 2c,d).

### Mitochondrial dynamics affect PME

**Figure 1:**
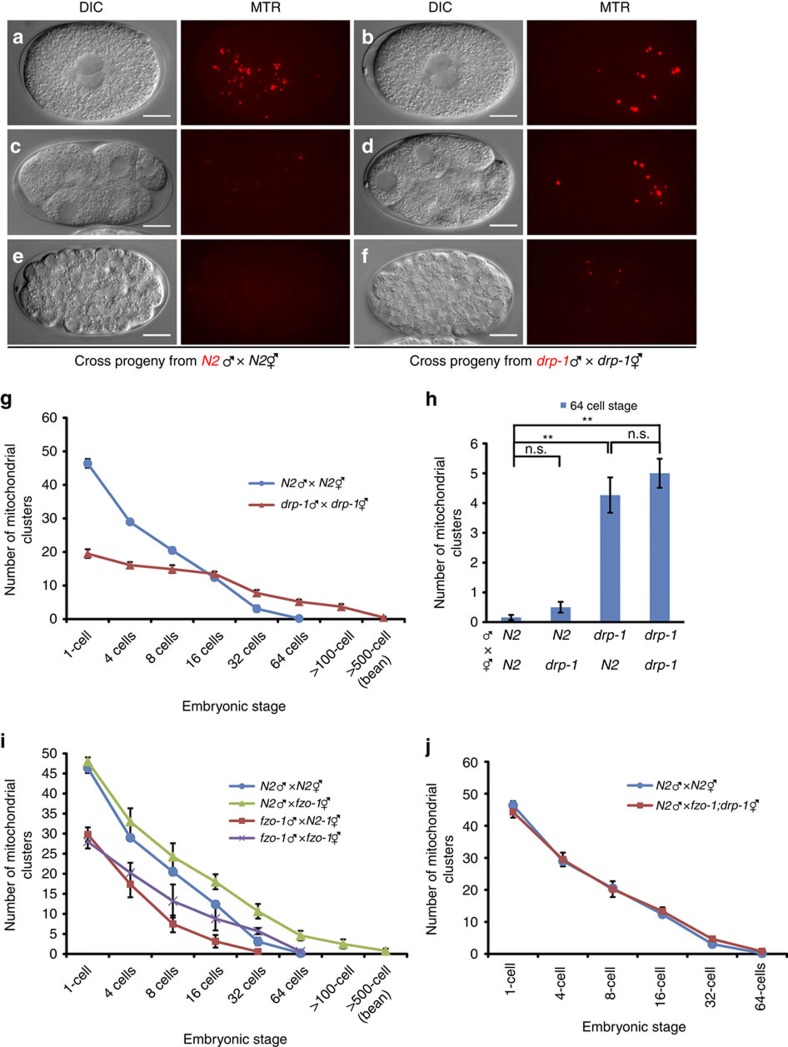
Mitochondrial dynamics regulate PME. (**a**–**f**) Delayed PME caused by a defect in paternal mitochondrial fission. Differential interference contrast (DIC) and MitoTracker Red (MTR) images of cross-fertilized embryos from mating of MTR-stained N2 males or *drp-1(tm1108)* males with unstained N2 or *drp-1(tm1108)* hermaphrodites are shown. Red dots are mitochondrial clusters stained by MTR. The stages of embryos shown are: 1-cell embryos at the pronuclear-meeting stage (**a**,**b**), 8-cell stage embryos (**c**,**d**), and approximately 100-cell stage embryos (**e**,**f**). Scale bars represent 10 μm. (**g**,**i**,**j**) The numbers of MTR-stained paternal mitochondrial clusters in cross-fertilized embryos from the indicated crosses between MTR-stained males and unstained hermaphrodites were scored at different stages of embryos (1-cell, 4-cell, 8-cell, 16-cell, 32-cell, 64-cell, >100-cell and bean stages). Data shown are mean±s.e.m. (*n*=10). (**h**) The numbers of MTR-stained paternal mitochondrial clusters in 64-cell stage cross-fertilized embryos from the indicated crosses were scored as in **g**. Data shown are mean±s.e.m. (*n*=15). The significance of difference between different mating experiments was determined by unpaired *t* test. **P<0.01. ‘n.s.' indicates no significant difference.

We first examined, using fluorescence microscopy, the fate of sperm mitochondria in cross-fertilized embryos from parents that are both defective in *drp-1* (refs 9, 10, 11). In these experiments, MitoTracker Red (MTR), a mitochondrial specific dye, was used to stain sperm mitochondria, which generated a mitochondrial staining pattern identical to that of the WAH-1::GFP fusion, a known mitochondrial protein (Supplementary Fig. 3a)^34^,^35^. N2 or *drp-1(tm1108)* males, prestained with MTR, were mated with unstained N2 or *drp-1(tm1108)* hermaphrodites. Fertilized eggs were dissected from the mated hermaphrodites and observed by fluorescence microscopy. Compared with cross-fertilized embryos from mating between N2 males and hermaphrodites, embryos from mating between *drp-1(tm1108)* parents had larger and brighter MTR-stained paternal mitochondrial clusters, but fewer in number (Fig. 1a,b). This observation is consistent with the EM analysis of mitochondria in N2 and *drp-1(tm1108)* sperm. Although the number of MTR-stained paternal mitochondrial clusters in *drp-1(tm1108)* zygotes was less than that in N2 zygotes (Fig. 1g), they persisted substantially longer (until approximately the 500-cell embryonic stage) than those in N2 cross-fertilized embryos, which disappeared before the 100-cell embryonic stage (Fig. 1a–g). This result suggests that a defect in mitochondrial fission delays PME. We then examined if proper mitochondrial fission is required maternally, paternally or both to promote PME, by crossing MTR-stained N2 males into *drp-1(tm1108)* hermaphrodites or MTR-stained *drp-1(tm1108)* males into N2 hermaphrodites. We counted the number of MTR-stained paternal mitochondrial clusters in 64-cell stage cross-fertilized embryos, before the initiation of most zygotic gene expression, which could complicate the analysis of paternal and maternal contributions^36^. Cross-fertilized embryos from N2 hermaphrodites mated with *drp-1(tm1108)* males had similar numbers of MTR-stained paternal mitochondrial clusters to those seen in cross progeny between *drp-1(tm1108)* parents (Fig. 1g,h). By contrast, few MTR-stained paternal mitochondria were seen in 64-cell stage cross-fertilized embryos from mating of *drp-1(tm1108)* hermaphrodites with N2 males (Fig. 1h). These results indicate that larger, elongated paternal mitochondria from *drp-1(tm1108)* males are removed less efficiently than smaller and rounded paternal mitochondria from N2 males and that a maternal mitochondrial fission defect does not affect PME.

Using a similar strategy, we investigated the roles of mitochondrial fusion and the profusion gene *fzo-1* in PME. Interestingly, we observed contrasting outcomes, depending on the parental genetic backgrounds. Paternal mitochondria were removed slightly faster in cross-fertilized embryos from N2 hermaphrodites mated with MTR-stained *fzo-1(tm1133)* males and disappeared by the 32-cell embryonic stage (Fig. 1i). By contrast, the rate of PME was substantially reduced in cross-fertilized embryos from mating of *fzo-1(tm1133)* hermaphrodites with MTR-stained N2 males, leading to persistence of paternal mitochondria until the 500-cell embryonic stage (Fig. 1i). In cross-fertilized embryos between MTR-stained *fzo-1(tm1133)* males and unstained *fzo-1(tm1133)* hermaphrodites, the opposite paternal and maternal effects on PME caused by the *fzo-1(tm1133)* mutation neutralized each other and paternal mitochondria disappeared by the 64-cell stage embryo as in N2 cross-fertilized embryos (Fig. 1i). Therefore, paternal mitochondria from *fzo-1(tm1133)* males are removed faster in embryos, whereas excessive maternal mitochondrial fission in *fzo-1(tm1133)* embryos, as have been demonstrated previously^25^,^26^, delays PME. Consistent with this observation, in cross-fertilized embryos between MTR-stained N2 males and unstained *fzo-1(tm1133); drp-1(tm1108)* hermaphrodites, in which loss of *drp-1* suppresses *fzo-1(tm1133)*-induced mitochondrial fragmentation and restores tubular mitochondrial network (Supplementary Fig. 1b–e)^25^, paternal mitochondria were eliminated at the same rate as that seen in cross-progeny between N2 males and hermaphrodites (Fig. 1j).

### Loss of *fzo-1* impairs autophagosome formation

**Figure 2:**
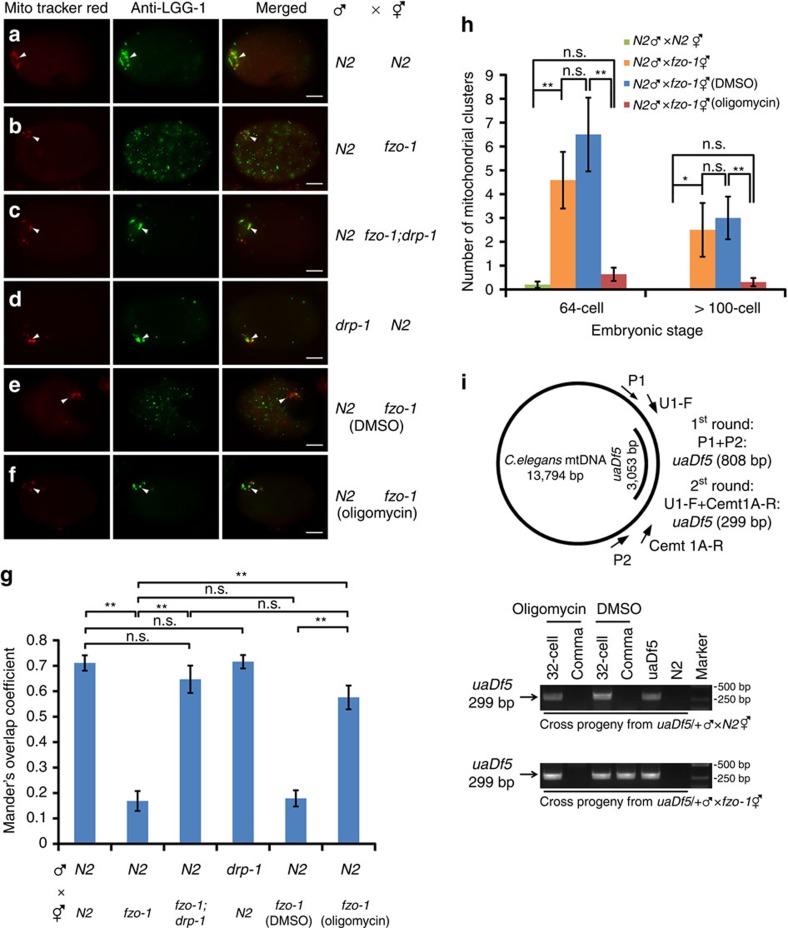
Compromised maternal mitochondria in *fzo-1(tm1133)* oocytes compete with paternal mitochondria for autophagosomes. (**a**–**f**) Analysis of colocalization of LGG-1 autophagosomes with MTR-stained paternal mitochondrial clusters in zygotes. Zygotes from mating of MTR-stained males with unstained hermaphrodites right after sperm entry were fixed and stained with an antibody to LGG-1. Arrowheads indicate MTR-stained paternal mitochondrial clusters, some of which were colocalized with LGG-1 aggregates. Scale bar represents 10 μm. (**g**) Colocalization analysis using Mander's overlap coefficient (MOC). MOC (with thresholds) represents the degree of colocalization between MTR-stained paternal mitochondrial clusters (Red) and LGG-1 aggregates (Green). The significance of difference between different mating experiments was determined by unpaired *t* test. **P<0.01. ‘n.s.' indicates no significant difference. 15 zygotes from each mating were scored. (**h**) Oligomycin treatment suppresses the defect of delayed PME caused by loss of maternal *fzo-1*. Quantification of MTR-stained paternal mitochondrial clusters in 64-cell and>100-cell stage cross-fertilized embryos from the indicated crosses grown on regular Nematode Growth Medium (NGM) plates or NGM plates with the DMSO control or 150 μg ml^−1^ oligomycin was performed as in Fig. 1g. In all crosses, MTR-stained males were mated with unstained hermaphrodites. Data shown are mean±s.e.m. (*n*=10). The significance of difference between different mating experiments was determined by unpaired *t* test. *P<0.05, **P<0.01. ‘n.s.' indicates no significant difference. (**i**) PCR-based assays to monitor persistent *uaDf5* paternal mtDNA in cross-fertilized embryos. Upper panel, a schematic diagram depicts the *C. elegans* mitochondrial genome and the *uaDf5* deletion. The primers used in the nested PCR assays to amplify the *uaDf5* deletion and the expected sizes of PCR products are indicated. These primers failed to amplify wild-type mtDNA (expected size of 3861, bp) under the PCR conditions used. Bottom panel, the results of PCR analysis of 32-cell and comma stage cross-fertilized embryos from mating of MTR-stained *uaDf5/+* males with unstained *fzo-1(tm1133)* or N2 hermaphrodites grown on NGM plates containing either the DMSO control or 150 μg ml^−1^ oligomycin. Each PCR reaction was prepared from eight cross-fertilized embryos. In all panels, *fzo-1(tm1133)* and *drp-1(tm1108)* alleles were used.

The observation that a defect in maternal mitochondrial fusion delays PME, which is suppressed by a maternal mitochondrial fission defect, is intriguing. One possibility is that excessive maternal mitochondrial fragmentation in *fzo-1(tm1133)* embryos may generate an increased number of compromised maternal mitochondria^16^,^21^, which is evidenced by their poor staining by TMRE (Supplementary Fig. 1a,c,d), a mitochondrial membrane potential sensitive dye^37^, and which may end up competing with paternal mitochondria for interactions with the autophagy machinery. We examined the localization pattern of LGG-1, a *C. elegans* homolog of LC3/Atg8 that is required for autophagosome formation^12^,^38^, using a monoclonal antibody to LGG-1^39^. In zygotes between MTR-stained N2 males and unstained N2 hermaphrodites, bright LGG-1 staining was seen clustering around MTR-stained paternal mitochondria near the site of sperm entry (Fig. 2a). We quantified the degree of the colocalization between MTR paternal mitochondrial clusters and LGG-1 puncta using Mander's overlap coefficient (MOC) and found that the MOC is above 0.70 (Fig. 2g), indicating good colocalization between paternal mitochondrial clusters and LGG-1 puncta. By contrast, in zygotes from mating between *fzo-1(tm1133)* hermaphrodites and MTR-stained N2 males, LGG-1 existed as dispersed puncta throughout the embryos (Fig. 2b) and the MOC is less than 0.17 (Fig. 2g), indicating poor colocalization. Since we observed similar dispersed LGG-1 puncta in unfertilized *fzo-1(tm1133)* oocytes but much less LGG-1 puncta in unfertilized N2 oocytes (Supplementary Fig. 4a,b,f), the formation of these dispersed LGG-1 puncta or autophagosomes is independent of fertilization and indicates the presence of many damaged organelles in *fzo-1(tm1133)* oocytes. Consistent with the observation that loss of *drp-1* suppresses excessive mitochondrial fission in *fzo-1(tm1133)* embryos (Supplementary Fig. 1c–e)^25^, and potentially, the associated mitochondrial damages, the number of dispersed LGG-1 puncta was greatly reduced in *fzo-1(tm1133); drp-1(tm1108)* fertilized or unfertilized oocytes (Fig. 2c, Supplementary Fig. 4c,f). In *fzo-1(tm1133); drp-1(tm1108)* fertilized eggs, most of the MTR-stained paternal mitochondria from N2 males are colocalized with LGG-1 autophagosomes, with a 0.65 MOC (Fig. 2c,g). Although paternal mitochondria from *drp-1(tm1108)* males were removed less efficiently than those from N2 males in N2 fertilized eggs (Fig. 1a–h), most of the *drp-1(tm1108)* sperm mitochondria are colocalized with LGG-1 autophagosomes, with a 0.72 MOC (Fig. 2d,g).

**Figure 3:**
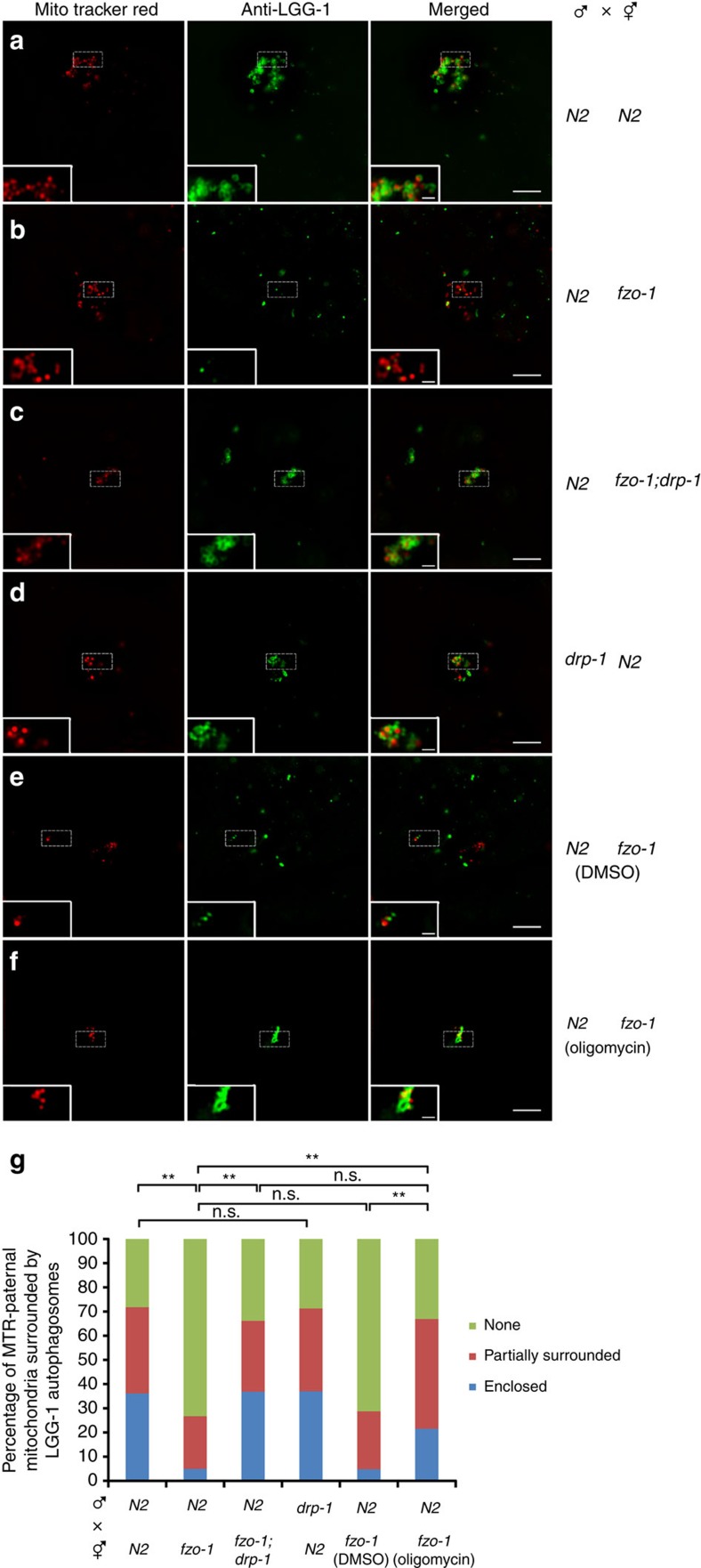
Structured illumination microscopy (SIM) analysis of paternal mitochondria surrounded by autophagosomes in zygotes. (**a**–**f**) Analysis of MTR-stained paternal mitochondria surrounded by LGG-1 autophagosomes in zygotes. In all panels, zygotes from mating of MTR-stained males and unstained hermaphrodites with the indicated genotypes right after sperm entry were stained with an antibody to LGG-1 as described in Fig. 2. Scale bar represents 5 μm. A higher magnification view of MTR-stained paternal mitochondria and LGG-1 autophagosomes is shown in the inset of each image panel. Scale bars in the insets are 1 μm. (**g**) Percentages of MTR-stained paternal mitochondria enclosed or partially surrounded by LGG-1 autophagosomes in the indicated crosses are shown. MTR-stained paternal mitochondria not in direct contact with any LGG-1 puncta were scored as a ‘none' (not surrounded by an autophagosome). The significance of difference between different mating experiments was determined by Chi-square test. **P<0.01. ‘n.s.' indicates no significant difference. The numbers of MTR-stained paternal mitochondria scored are 202 (**a**), 202 (**b**), 133 (**c**), 108 (**d**), 146 (**e**) and 130 (**f**), respectively. In all panels, *fzo-1(tm1133)* and *drp-1(tm1108)* alleles were used.

We then performed structured illumination microscopy (SIM) to analyze with greater resolution the interactions between paternal mitochondria and autophagosomes in embryos. In zygotes from mating between MTR-stained N2 males and unstained N2 hermaphrodites, more than 70% of MTR-stained paternal mitochondria were enclosed or partially surrounded by LGG-1 autophagosomes (Fig. 3a,g), whereas in zygotes from mating between unstained *fzo-1(tm1133)* hermaphrodites and MTR-stained N2 males, 73% of MTR-stained paternal mitochondria were away from the LGG-1 puncta (Fig. 3b,g). Consistent with the observations above (Fig. 2c), loss of *drp-1* restored enclosure or partial wrapping of MTR-stained paternal mitochondria by LGG-1 autophagosomes in *fzo-1(tm1133)* oocytes to the level seen in N2 embryos (Fig. 3c,g). Moreover, a high percentage (70%) of *drp-1(tm1108)* sperm mitochondria were completely or partially surrounded by autophagosomes upon their entry into N2 oocytes (Fig. 3d,g), indicating that the long and bulky paternal mitochondria from *drp-1(tm1108)* sperm are recognized and surrounded normally by autophagosomes, but may deter subsequent degradation steps after autophagosome recruitment because of the larger mitochondrial sizes.

### Paternal mitochondria are permeabilized after fertilization

To better understand how paternal mitochondria are selectively targeted for degradation in embryos, we used TMRE, a membrane-potential sensitive dye that can be sequestered only by active mitochondria^37^, and MitoTracker Green, a mitochondrial dye that stains mitochondria regardless of the mitochondrial membrane potential, to label N2 sperm mitochondria, and then mated the stained N2 males with unstained N2 hermaphrodites to observe the integrity of paternal mitochondria before and after fertilization. Although both TMRE and MitoTracker Green strongly labelled mitochondria in spermatozoa (Supplementary Fig. 3b), only MitoTracker Green staining was detected in paternal mitochondria in fertilized oocytes and TMRE signals were completely lost (Supplementary Fig. 3c), indicating that paternal mitochondria lose their membrane potential after entry into the oocyte.

**Figure 4:**
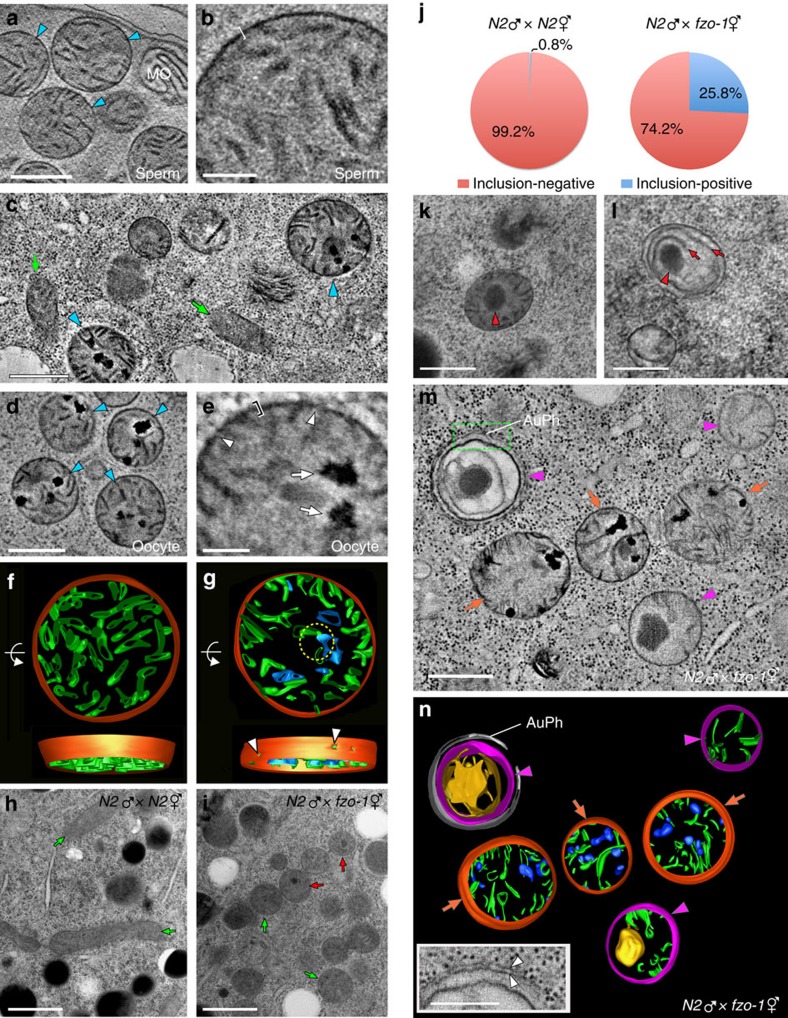
Electron tomography (ET) analyses of mitochondria in spermatozoa and embryos. (**a**,**b**) An ET slice image of mitochondria in an N2 spermatozoon (**a**). Mitochondria are enclosed by smooth and intact membranes (bracket in **b**). MO: membranous organelle. (**c**) An ET image of the cytoplasm of an N2 zygote. Maternal (arrows) and paternal (arrowheads) mitochondria are indicated. (**d**,**e**) An ET slice image of sperm mitochondria in an N2 zygote (**d**). Their mitochondrial membranes appear fuzzy (bracket) and have cracks (arrowheads) and their matrices contain electron dense spots (arrows) distinctive of paternal mitochondria (**e**). (**f**,**g**) 3D ET models of paternal mitochondria in an N2 spermatozoon (**f**) and an N2 zygote (**g**), generated from tomograms shown in **b** and **e**, respectively. Arrowheads in **g** mark punctures in the mitochondrial membranes seen as cracks in **e**. Mitochondrial membranes, cristae and speckles are coloured in red, green and blue, respectively. (**h**,**i**) Electron micrographs of fertilized eggs from the indicated crosses. Round mitochondria in the *fzo-1(tm1133)* egg contrast with elongated mitochondria in the N2 egg (arrows). Some *fzo-1(tm1133)* maternal mitochondria have a dark inclusion (red arrows). The dark spherical objects in **h** are lipid droplets. (**j**) Pie charts showing ratios of inclusion-positive to inclusion-negative maternal mitochondria in N2 (*n*=392) and *fzo-1* (*n*=446) fertilized eggs. (**k**,**l**) Aberrant maternal mitochondria in *fzo-1(tm1133)* embryos with dark inclusions (arrowheads). Some of their cristae transform into long lamellae (arrows in **l**). (**m**,**n**) An ET slice image (**m**) of the cytoplasm of a fertilized *fzo-1(tm1133)* egg and 3D models of the mitochondria in **m** (**n**). Maternal (magenta arrowheads) and paternal (orange arrows) mitochondria in *fzo-1(tm1133)* eggs are easily differentiated by their distinctive features of electron-dense patches and cristae. One maternal mitochondrion is enclosed by an autophagosome (AuPh). A high magnification view of Auph and the maternal mitochondrion (green rectangle in **m**) is shown in the inset in **n**. The blue blobs in paternal mitochondria indicate the electron dense patches in **m**. The double membranes of the autophagosome are marked with arrowheads in the inset. Scale bars indicate 500 nm (**a**,**c**,**d**,**h**,**i**,**k**–**m**) or 100 nm (**b**,**e** and inset in **n**).

We then compared high-resolution ultrastructural images of sperm mitochondria before and after fertilization using electron tomography (ET). As discussed above, mitochondria in N2 sperm are spherical (Fig. 4a, Supplementary Fig. 2a and Supplementary Movie 1), with an average diameter of 513 nm (s.e.m. 10 nm). Paternal mitochondria (blue arrowheads in Fig. 4c,d) in the zygote can be easily distinguished from maternal mitochondria because the latter are tubular and thinner (with an average width of 238 nm and s.e.m. 9 nm; indicated by green arrows in Fig. 4c)^26^. In spermatozoa, mitochondria have flat cristae that uniformly spread out in the matrix (Fig. 4a,b) and the surface membranes of mitochondria constitute a smooth outline with an average thickness of 11 nm (bracket, Fig. 4b and Supplementary Fig. 5a,i). By contrast, the surface membranes of paternal mitochondria in the zygote appear irregular (bracket, Fig. 4e and Supplementary Fig. 5b) and have discontinuities that constitute holes (arrowheads, Fig. 4e). The average thickness of the paternal mitochondrial membranes in the tomograms increases to 18 nm after fertilization (Supplementary Fig. 5i). Another abnormality that occurs in paternal mitochondria in the zygote is the condensed speckles scattered in their matrix (Fig. 4d, arrows in Fig. 4e, and Supplementary Fig. 5c,d)^40^. As reported previously^11^,^40^, some of the paternal mitochondria were surrounded by double-membrane autophagosomes (Supplementary Fig. 5d,h). In later stage embryos, paternal mitochondria either have larger speckles or lose most of the matrix contents inside an enclosed autophagosome (Supplementary Fig. 5d,e).

Three-dimensional (3D) reconstruction of mitochondrial membranous elements reveals that cristae become shrivelled as the speckles grow and that the dark speckles are connected to the cristae (Fig. 4f,g), suggesting that cristae of paternal mitochondria collapse after fertilization. Moreover, multiple holes are clearly observed on the surface of paternal mitochondria in zygotes, which are not seen in mitochondria from spermatozoa (Fig. 4f,g). These results indicate that the membranes of paternal mitochondria are permeabilized and depolarized and that their cristae collapse into speckles and are damaged upon entry into the oocyte, which likely triggers elimination of paternal mitochondria by mitophagy^41^.

### Compromised maternal mitochondria in *fzo-1(tm1133)* embryos

**Figure 5:**
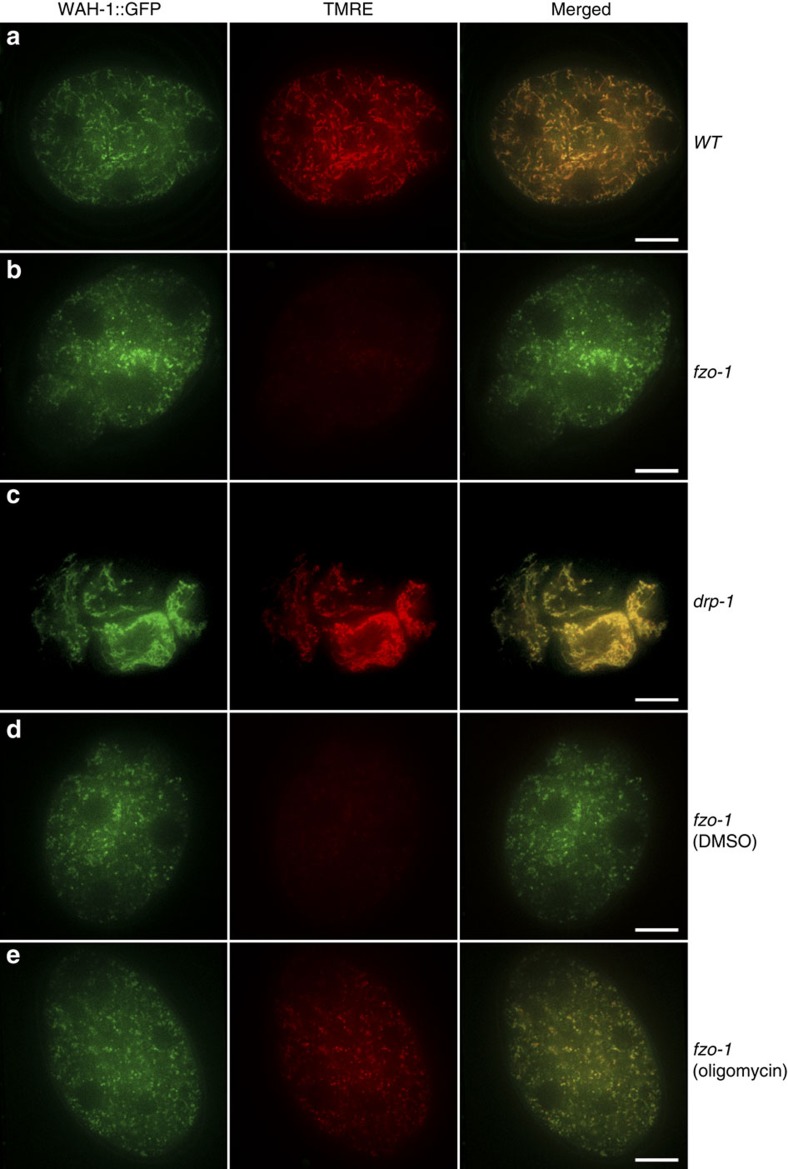
Analyses of mitochondrial membrane potential in maternal mitochondria from different strains. TMRE staining of 4-cell stage embryos from the following strains are shown: wild type (**a**), *fzo-1(tm1133)* (**b**), *drp-1(tm1108)* (**c**), and *fzo-1(tm1133)* animals treated with DMSO (**d**) or 150 μg ml^−1^ oligomycin (**e**). All strains contain the *wah-1::gfp* knock-in allele, which was used as an internal mitochondrial marker that labelled both normal and compromised maternal mitochondria. Confocal images of WAH-1::GFP, TMRE and WAH-1::GFP/TMRE merged from embryos dissected from hermaphrodites prestained with TMRE are shown. The exposure time, laser strength and other parameters in each channel are identical for all embryos. The exposure time for the 488 nm laser is 100 ms and for the 561 nm laser is 30 ms. Scale bar represents 10 μm.

The presence of many dispersed LGG-1 puncta in unfertilized *fzo-1(tm1133)* oocytes, which are largely absent in unfertilized *fzo-1(tm1133); drp-1(tm1108)* oocytes (Supplementary Fig. 4b,c,f), suggests that maternal mitochondria are compromised in *fzo-1(tm1133)* oocytes. We used the membrane potential-sensitive dye TMRE to stain maternal mitochondria in embryos from different genetic backgrounds that also contain the *wah-1::gfp* knock-in. Bright and tubule TMRE staining was seen in N2 embryos and completely overlapped with that of WAH-1::GFP (Fig. 5a). In *fzo-1(tm1133)* embryos, the TMRE staining was much weaker (Fig. 5b), while the WAH-1::GFP fluorescence remained strong, but exhibited punctiform staining, indicating that mitochondria in *fzo-1(tm1133)* embryos are fragmented and depolarized. In *drp-1(tm1108)* embryos, strong and asymmetric TMRE staining appeared as highly connected clusters and also completely overlapped with that of WAH-1::GFP (Fig. 5c), which is consistent with a defect in mitochondrial fission.

We performed ET analysis of embryos from mating of N2 males with N2 or *fzo-1(tm1133)* hermaphrodites to understand how maternal loss of *fzo-1* delays PME. As described previously^25^,^26^, most maternal mitochondria in embryos from mating of N2 males and hermaphrodites displayed a long, tubular morphology (green arrows; Fig. 4h). By contrast, maternal mitochondria in *fzo-1(tm1133)* fertilized eggs are highly fragmented and spherical (green and red arrows; Fig. 4i). Interestingly, about 26% of maternal mitochondria in *fzo-1(tm1133)* fertilized eggs had an electron-dense inclusion in the matrix (red arrows; Fig. 4i,j). Those mitochondria with the inclusions had reduced numbers of cristae (Fig. 4k), which appeared to merge into lamellae in the matrix (red arrows; Fig. 4l). Similar spherical mitochondria with dark inclusions were seen in somatic cells of *fzo-1(tm1133)* animals (Supplementary Fig. 5f,g). This kind of compromised maternal mitochondria are rarely observed (0.8%) in N2 embryos (Fig. 4h,j) and are easily distinguished from paternal mitochondria by ET (Fig. 4m,n and Supplementary Movie 2). In the matrix of defective *fzo-1(tm1133)* maternal mitochondria, a large dense inclusion was often surrounded by extended lamellae (Fig. 4l,m), whereas paternal mitochondria contained multiple small dark speckles and their cristae shrank (Fig. 4c–e,g,m,n). Like damaged paternal mitochondria that were often surrounded by autophagosomes (Supplementary Fig. 5d,e,h), some aberrant *fzo-1(tm1133)* maternal mitochondria were also enclosed by double-membrane autophagosomes (Fig. 4m and inset in 4n), indicating that they are compromised mitochondria undergoing mitophagy. These results indicate that defective *fzo-1(tm1133)* maternal mitochondria can compete with paternal mitochondria for the autophagy machinery and thus delay PME.

**Figure 6:**
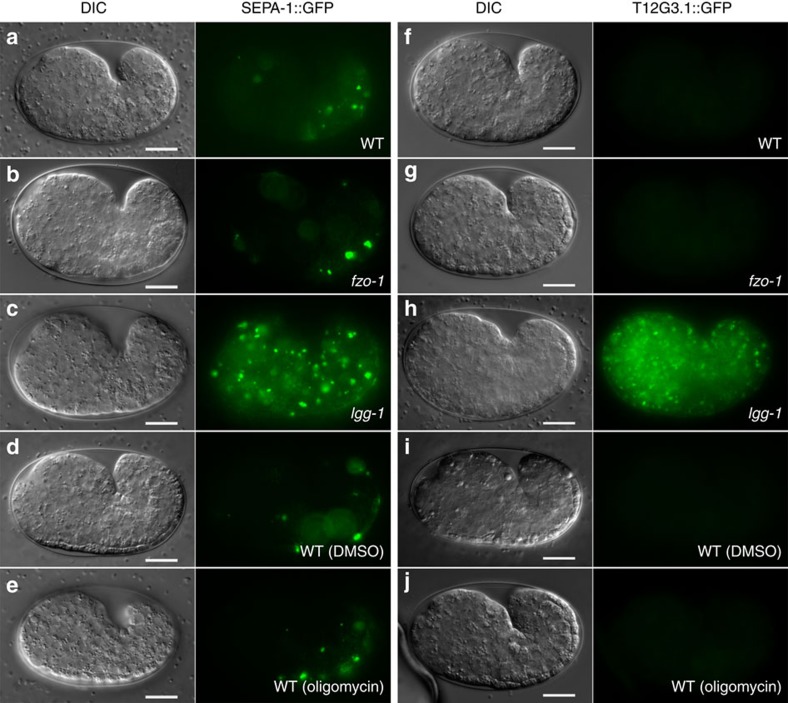
Loss of *fzo-1* and oligomycin treatment do not affect normal autophagic degradation during embryogenesis. Expression patterns of SEPA-1::GFP (**a**–**e**) or T12G3.1::GFP (**f**–**j**) in comma-stage embryos of the indicated strains are shown. Embryos examined are: wild-type (**a**,**f**), *fzo-1(tm1133)* (**b**,**g**), *lgg-1(bp500)* (**c**,**h**) wild-type animals treated with DMSO (**d**,**i**) or 150 μg ml^−1^ oligomycin (**e**,**j**). Exposure time is 1 s for SEPA-1::GFP and 2 s for T12G3.1::GFP. Scale bar represents 10 μm.

It is possible that compromised maternal mitochondria in *fzo-1(tm1133)* embryos cause reduced mitochondrial functions and ATP production, which may impair the autophagy process. We thus examined if loss of *fzo-1* affects autophagic degradation of the SEPA-1::GFP fusion and the T12G3.1::GFP fusion^39^, both of which are preferentially removed by autophagy during embryogenesis. In N2 and *fzo-1(tm1133)* embryos, both SEPA-1::GFP and T12G3.1::GFP were barely visible (Fig. 6a,b,f,g), indicating that they were degraded efficiently by autophagy. By contrast, in *lgg-1(bp500)* embryos that are defective in autophagy, the expression levels of both GFP fusions were greatly increased and widespread GFP aggregates were observed (Fig. 6c,h). Therefore, the autophagy process is not compromised in the *fzo-1(tm1133)* mutant and delayed PME is likely a result of competition for the autophagy machinery between compromised maternal mitochondria and damaged paternal mitochondria.

### Oligomycin rescues the PME defect in *fzo-1(tm1133)* embryos

Because the mitochondrial fission process can trigger mitophagy by reducing the membrane potential of some of the daughter mitochondria^21^, we examined if increasing the membrane potential of compromised maternal mitochondria in *fzo-1(tm1133)* embryos could prevent targeting of maternal mitochondria by the autophagy machinery and thus enhance autophagosome recruitment to paternal mitochondria and PME. We used oligomycin, an inhibitor of ATP synthase, to increase the membrane potential of mitochondria (oligomycin acts by blocking proton translocation to the mitochondrial matrix)^42^. As expected, treatment of *fzo-1(tm1133)* animals with oligomycin significantly enhanced TMRE staining of *fzo-1(tm1133)* embryos (Fig. 5b,e), whereas *fzo-1(tm1133)* embryos treated with the dimethylsulfoxide (DMSO) control remained poorly stained by TMRE (Fig. 5d). Since mitochondria in *fzo-1(tm1133)* embryos remained fragmented after oligomycin treatment (Fig. 5e), these results indicate that oligomycin treatment increases the membrane potential of *fzo-1(tm1133)* mitochondria but does not block excessive mitochondrial fission. Oligomycin treatment did not affect the autophagy process either, as autophagic degradation of SEPA-1::GFP and T12G3.1::GFP occurred normally in oligomycin-treated embryos (Fig. 6d,e,i,j).

We then mated MTR-stained N2 males with *fzo-1(tm1133)* hermaphrodites grown on plates with or without oligomycin. On plates without oligomycin (DMSO control), PME was delayed significantly, similar to that seen in cross-progeny between N2 males and *fzo-1(tm1133)* hermaphrodites grown on regular plates (Fig. 2h). On plates with oligomycin, paternal mitochondria were removed significantly faster, at a rate very close to that seen in mating between N2 males and hermaphrodites (Fig. 2h), indicating that treatment with oligomycin suppresses the PME defect caused by *fzo-1(tm1133)*. We corroborated these results using a polymerase chain reaction (PCR)-based assay, in which the fate of paternal mitochondria carrying a 3053-bp mtDNA deletion (*uaDf5*) can be tracked in embryos by PCR analysis^9^,^10^,^11^. When N2 males heteroplasmic with *uaDf5* (*uaDf5/+*) were mated with N2 hermaphrodites on plates with the DMSO control, paternal *uaDf5* mtDNA was still visible in the 32-cell stage embryos, but disappeared in embryos beyond 100-cell stage, including the comma-stage embryos at around 500 cells (Fig. 2i), as they did in the same mating on regular plates^9^. Treatment with oligomycin in the same mating experiment did not alter the rate of PME (Fig. 2i and Supplementary Fig. 4g), indicating that oligomycin treatment cannot alleviate damages to paternal mitochondria in embryos to prevent or delay PME. When *uaDf5/+*males were mated with *fzo-1(tm1133)* hermaphrodites on the DMSO control plates, *uaDf5* paternal mtDNA persisted significantly longer and was still visible in the comma stage embryos (Fig. 2i). However, when the same mating was done on plates with oligomycin, *uaDf5* paternal mtDNA was no longer detected in comma stage embryos (Fig. 2i), indicating that oligomycin treatment accelerates removal of paternal mitochondria in *fzo-1(tm1133)* oocytes.

Consistent with the observations that oligomycin treatment could rescue the PME defect in *fzo-1(tm1133)* embryos, when MTR-stained N2 males were mated with *fzo-1(tm1133)* hermaphrodites on oligomycin plates, substantial colocalization of MTR-stained paternal mitochondrial clusters with LGG-1 puncta was observed, with a MOC of 0.58, compared with a 0.18 MOC in such colocalization when the same mating was done on the DMSO control plates (Fig. 2e–g). Similarly, high resolution SIM microscopy showed that 67% of MTR-stained paternal mitochondria were completely or partially surrounded by LGG-1 autophagosomes in zygotes from mating of N2 males with *fzo-1(tm1133)* hermaphrodites grown on oligomycin plates, compared with 29% of MTR paternal mitochondria completely or partially surrounded by autophagosomes when the same mating was done on the DMSO control plates (Fig. 3e–g). Moreover, significantly less LGG-1 puncta were observed in unfertilized *fzo-1(tm1133)* oocytes treated by oligomycin than those treated with the DMSO control (Supplementary Fig. 4d–f). Taken together, these results provide strong support to the model that excessive maternal mitochondrial fission increases the number of compromised maternal mitochondria with reduced membrane potential, which compete for the autophagy machinery and lead to inefficient recruitment of autophagosomes to paternal mitochondria and delayed PME. These results also provide the mechanistic basis for how paternal mitochondria are distinguished from maternal mitochondria and selectively targeted for autophagic degradation.

### Combined impact of mitochondrial fusion and fission to PME

**Figure 7:**
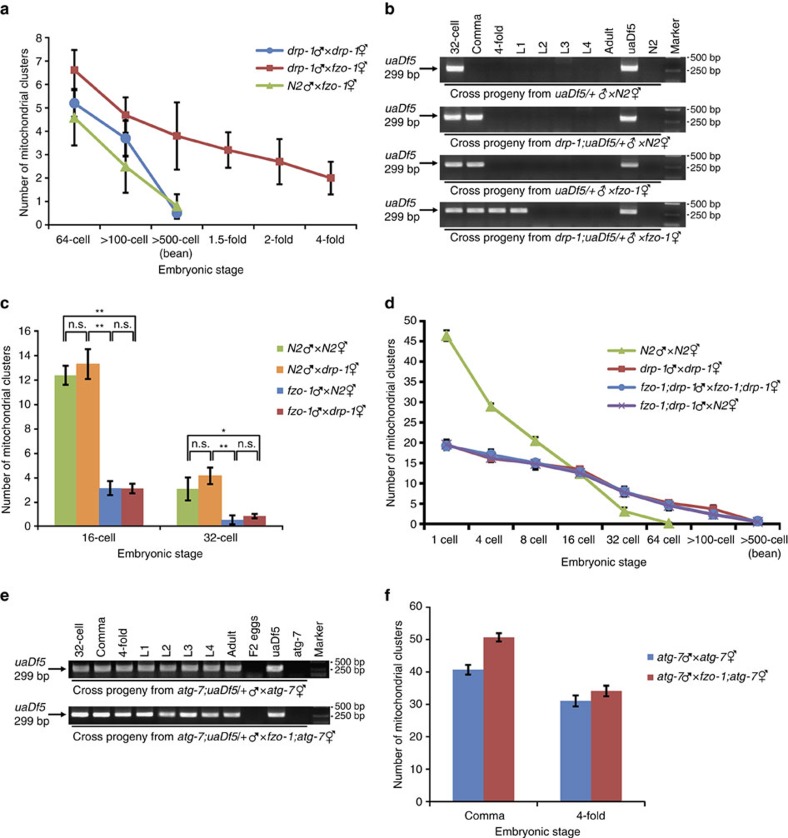
A synergistic effect of a paternal mitochondrial fission defect and a maternal mitochondrial fusion defect in delaying PME. (**a**,**d**) Quantification of MTR-stained paternal mitochondrial clusters in different stages of cross-fertilized embryos from the indicated crosses. In all cases, MTR-stained males were mated with unstained hermaphrodites. Data shown are mean±s.e.m. (*n*=10). (**b**,**e**) PCR-based assays to monitor paternal *uaDf5* mtDNA in different stages of cross-fertilized embryos or animals from the indicated crosses were performed as in Fig. 2i. Each PCR assay was prepared from eight cross-fertilized embryos or animals (see Methods). Uncropped gel images are shown in Supplementary Fig. 7. (**c**,**f**) Quantification of MTR-stained paternal mitochondrial clusters in 16- and 32-cell stage (**c**) or comma and 4-fold stage (**f**) cross-fertilized embryos from the indicated crosses. Data shown are mean±s.e.m. (*n*=15). MTR-stained males were mated with unstained hermaphrodites (**a**,**c**,**d**,**f**). The significance of difference between different mating experiments was determined by unpaired *t* test. *P<0.05, **P<0.01. ‘n.s.' indicates no significant difference. In all panels, *atg-7(bp422), drp-1*(*tm1108*) and *fzo-1*(*tm1133*) alleles were used.

We examined the combined impact of the mitochondrial fusion and fission processes to PME. In cross progeny from *fzo-1(tm1133)* hermaphrodites mated with MTR-stained *drp-1(tm1108)* males, PME was greatly delayed and MTR-stained paternal mitochondria were observed in all stages of embryogenesis (Fig. 7a). In PCR-based assays, when *drp-1(tm1108)*; *uaDf5/+* males were mated with N2 hermaphrodites or when *uaDf5/+* males were mated with *fzo-1(tm1133)* hermaphrodites, *uaDf5* paternal mtDNA persisted significantly longer than it did in the cross between *uaDf5/+* males and N2 hermaphrodites and could still be detected in the comma embryos (Fig. 7b). When *drp-1(tm1108)*; *uaDf5/+* males were mated with *fzo-1(tm1133)* hermaphrodites, *uaDf5* paternal mtDNA persisted through all stages of embryogenesis and could be detected at the L1 larval stage (Fig. 7b), a further significant delay in removal of paternal mtDNA. These results indicate that a paternal mitochondrial fission defect synergizes with a maternal mitochondrial fusion defect to deter removal of paternal mitochondria. On the other hand, a paternal mitochondrial fusion defect caused by *fzo-1(tm1133)* accelerated PME in N2 oocytes to eliminate paternal mitochondria by the 32-cell stage embryos (Figs 1i and 7c), which was reversed by a *fzo-1(tm1133)* maternal mitochondrial fusion defect (Fig. 1i), but not by a *drp-1(tm1108)* maternal mitochondrial fission defect (Fig. 7c). The delay of PME was also less severe in cross-progeny between *fzo-1(tm1133)*; *drp-1(tm1108)* males and hermaphrodites, which showed a rate of PME similar to that seen in cross-progeny between *drp-1(tm1108)* males and hermaphrodites or between *fzo-1(tm1133)*; *drp-1(tm1108)* males and N2 hermaphrodites (Fig. 7d). These results further support the finding that a maternal mitochondrial fission defect is epistatic to the maternal mitochondrial fusion defect in affecting PME and that mitochondrial dynamics plays a critical role in regulating the kinetics of PME.

We last examined genetic interactions between mitochondrial dynamics and the autophagy process in regulating PME. The *C. elegans atg-7* gene encodes a homolog of the yeast Atg7 protein, which plays an important role in expansion and completion of the autophagosome^12^,^39^,^43^. We analysed the double mutant between *fzo-1(tm1133)* and a strong loss-of-function allele (*bp422*) of *atg-7*. When *atg-7(bp422)*; *uaDf5/+* males were mated with *atg-7(bp422)* hermaphrodites, *uaDf5* paternal mtDNA was detected in all stages of their cross progeny (F1 generation), from embryos, larvae, to adults, but was not found in the second generation (F2), indicating that paternal mtDNA persists through the life of the F1 cross progeny but does not pass to the next generation (Fig. 7e). *uaDf5* paternal mtDNA was also detected in all developmental stages of cross progeny between *atg-7(bp422)*; *uaDf5/+* males and *fzo-1(tm1133)*; *atg-7(bp422*) hermaphrodites and was similarly absent in the F2 generation (Fig. 7e). Quantification of the number of MTR-stained paternal mitochondrial clusters in cross progeny of *fzo-1(tm1133)*; *atg-7(bp422)* hermaphrodites or *atg-7(bp422)* hermaphrodites mated with MTR-stained *atg-7(bp422)* males showed that loss of both maternal *fzo-1* and *atg-7* resulted in retention of significantly more paternal mitochondria in comma stage embryos than loss of *atg-7* alone, but produced comparable numbers of paternal mitochondria in late stage 4-fold embryos (Fig. 7f). These results suggest that mitochondrial dynamics to some extent may act in parallel to the autophagy process to promote PME.

## Discussion

One of the long-standing puzzles in developmental biology is that mitochondria are inherited maternally in most animals^3^,^5^,^6^,^7^,^8^. How paternal mitochondria, but not maternal mitochondria, are selectively eliminated in early embryos is poorly understood and is a topic of great interest. It has been shown recently that the autophagic-lysosomal pathway plays an important role in removing paternal mitochondria in *C. elegans* embryos^9^,^10^,^11^. However, the mechanisms by which paternal mitochondria are distinguished from maternal mitochondria and ‘marked' for autophagic degradation in the fertilized egg remain largely unclear^40^.

In this study, we perform three-dimensional ET analyses of paternal mitochondria in spermatozoa and in fertilized eggs and find that paternal mitochondria, upon entry into the oocyte, are depolarized (Supplementary Fig. 3b,c), bear discontinuities and apertures on their surface (Fig. 4e,g), and lose a proportion of their cristae, which often appear crumpled and form multiple electron-dense speckles (Fig. 4d,e,g). These dramatic changes in the outer and inner structures of paternal mitochondria indicate that paternal mitochondria are damaged after their entry into the oocyte and thus become different from healthy maternal mitochondria in the zygote. Since depolarized or damaged mitochondria are normally targeted for autophagy^41^,^42^,^44^, these findings provide a simple and plausible mechanism by which paternal mitochondria are recognized and selectively degraded by the maternal autophagy machinery.

Consistent with this model, when maternal mitochondria are compromised due to excessive mitochondrial fission in mitochondrial fusion-defective embryos, the autophagy machinery, functioning normally in other developmental processes (Fig. 6a–c, f–h), is unable to distinguish the compromised maternal mitochondria from damaged paternal mitochondria and targets both for degradation (Fig. 4m,n and Supplementary Fig. 5h). This leads to reduced autophagosome formation on paternal mitochondria and less efficient PME. On the other hand, suppression of excessive mitochondrial fission by inactivating the pro-fission gene *drp-1* or by increasing the membrane potential of compromised maternal mitochondria through treatment with oligomycin markedly reduces the number of compromised maternal mitochondria and restores autophagosome formation on paternal mitochondria (Figs 2, 3 and 5), thereby restoring normal PME. These results provide strong supporting evidence to the model that paternal mitochondria are damaged following fertilization and are thus distinguished from normally healthy maternal mitochondria, leading to their selective recognition and removal by the maternal autophagy machinery.

**Figure 8:**
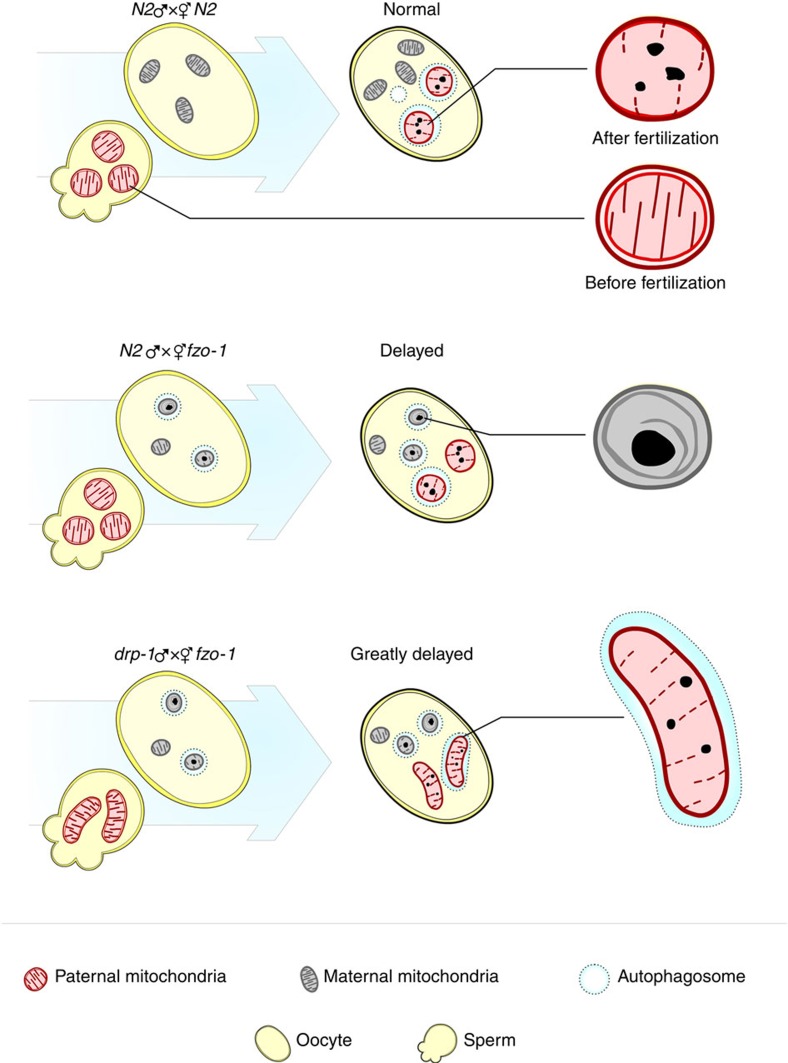
Diagrams summarizing the impacts of altered mitochondrial dynamics on the kinetics and specificity of PME. Maternal and paternal mitochondria are indicated with grey and red, respectively. Dash circles indicate autophagosomes.

Mitochondrial dynamics play an important role in mitochondrial quality control and stress response^16^,^21^,^22^,^23^,^45^. In this study, we discover a new and complex role of mitochondrial dynamics in regulating maternal mitochondrial inheritance. Specifically, a paternal mitochondrial fission defect slows down the removal of larger and elongated paternal mitochondria by autophagosomes^22^,^23^, whereas a paternal mitochondrial fusion defect accelerates elimination of paternal mitochondria, which may have already been compromised and become better targets for autophagosomes. Consistently, RNAi knockdown of the *eat-3* gene in N2 males, which encodes a homologue of the mammalian mitochondrial fusion protein OPA1^46^,^47^,^48^ and is important for mitochondrial fusion and for maintaining the cristae structures in *C. elegans*^26^,^27^, also accelerates PME (Supplementary Fig. 6). Although a maternal mitochondrial fission defect does not seem to affect PME, a maternal mitochondrial fusion defect causes excessive maternal mitochondrial fragmentation and an increased number of compromised maternal mitochondria, which compete for the autophagy machinery with damaged paternal mitochondria and lead to inefficient recruitment of autophagosomes to paternal mitochondria (Fig. 8). Moreover, a paternal mitochondrial fission defect synergizes with a maternal mitochondrial fusion defect to greatly delay PME. These new findings together provide important insights into the fundamental questions of how paternal mitochondria are selectively targeted for degradation by the autophagy machinery in early embryos and how mitochondrial dynamics play a critical and unexpected role in regulating the rate and specificity of PME.

## Methods

### *C. elegans* strains and culture conditions

We cultured *C. elegans* strains at 20 °C (ref. 49). Strains are maintained on Nematode Growth Medium (NGM) plates seeded with OP50, a uracil requiring mutant of *E. coli*. N2 is the wild-type strain. Alleles used in this study are: LGII, *fzo-1(tm1133)*; LGIV, *drp-1(tm1108), atg-7(bp422);* mtDNA*, uaDf5* (refs 26, 39, 50). All strains were backcrossed with N2 animals at least four times prior to analysis. *eat-3* RNAi treatment was carried out using a bacterial feeding protocol^51^. Briefly, animals were placed on NGM plates containing seeded bacteria expressing dsRNA specific for *eat-3.* L4 stage N2 males and hermaphrodites were grown and mated on RNAi plates for two generations. F2 adult males were then selected for mating experiments.

### MTR staining of paternal mitochondria

*C. elegans* males were grown overnight in the dark at 20 °C on NGM plates containing 4 μM MTR (Invitrogen, catalog number M7512), before being used in the mating experiments. MitoTracker Red stains mitochondria in live or fixed cells and displays red fluorescence.

### Microscopy and quantification of paternal mitochondria

Gonads of mated hermaphrodites derived from mating with MTR-stained males were dissected and oocytes or embryos were pushed out of the dissected gonads on an agarose pad. A coverslip was applied on top of the oocytes or embryos and M9 buffer (43.8 mM Na_2_HPO_4_, 22 mM KH_2_PO, 86 mM NaCl, 1 mM MgSO_4_) was pipetted into the empty space between the coverslip and the slide. Three dimensional (3D) images were acquired from the top to the bottom of the oocytes by a distance of 2 μm using a Zeiss Axio Imager M1 Nomarski microscope equipped with an AxioCam HRm CCD camera and AxioVision 4.6 software (Carl Zeiss Imaging Solutions GmbH, Inc.). Fluorescent images were deconvolved and projected into one plane. Mitochondrial clusters labelled by MTR were scored from deconvolved images of the dividing embryos at different embryonic stages.

### Antibody staining

Oocytes or embryos were pushed out of the dissected gonads of mated hermaphrodites and permeabilized and fixed using a freeze-crack method^52^. Briefly, embryos were placed in the M9 buffer on an adhesive glass slide. A coverslip was then placed on top of the embryos and slightly pressed to break open part of the embryo eggshells before putting the slide at −20 °C for 30 min. Embryos were fixed sequentially in methanol at −20 °C for 5 min and acetone at −20 °C for 5 min. Fixed embryos were blocked using PBF (PBS containing 10% fetal calf serum and 1% BSA) and stained with a monoclonal antibody to LGG-1 (provided by Hong Zhang at Institute of Biophysics, Chinese Academy of Sciences, 1:1000 dilution) at room temperature for 90 min. Following anti-LGG-1 staining, embryos were washed three times with PBS and stained with FITC-conjugated goat anti-mouse antibodies (CWBIO Inc., catalog number CW0113S, 1:50 dilution) at room temperature for 60 min. After that, embryos were washed with PBS and visualized using the Zeiss Axio Imager M1 Nomarski microscope equipped with an AxioCam HRm CCD camera or a structured illumination microscope (Nikon ECLIPSE Ti-E) equipped with NIS-element AR software (Laboratory Imaging) by taking 3D-images acquired from the top to the bottom of the embryo by a distance of 0.3 μm.

### MOC analysis

MOC analysis was performed using the Coloc 2 feature in Fiji image processing package. The background noises in the green and red channels were subtracted before any calculating. The total fluorescence intensity of the MTR-stained paternal mitochondrial clusters was quantified using the polygon selected ‘Region of interest (ROI)' tool. The MOC (with threshold) indicates the fraction of the total red fluorescence in ROI that overlaps with the green channel.

### Treatment of animals with oligomycin

Oligomycin (Sigma, catalog number O4876) was first dissolved in DMSO in a concentration of 2 mg ml^−1^, diluted to a final concentration of 150 μg ml^−1^, in M9 buffer,and then mixed with OP50 bacteria, which were spread on the NGM plates. Gravid hermaphrodites were grown and males and hermaphrodites were mated on these freshly made oligomycin plates overnight before hermaphrodites were dissected to obtain fertilized or unfertilized oocytes.

### TMRE and MitoTracker Green staining of sperm

MitoTracker Green (4 mM) and TMRE in DMSO (10 mM) were diluted to final concentrations of 600 and 100 μM, respectively, and then dribbled onto the OP50 bacterial lawn on NGM plates. Adult males were transferred to the NGM plates with TMRE and MitoTracker Green overnight in dark and then mated with unstained hermaphrodites or directly dissected to release sperm.

### TMRE staining of embryos and confocal microscopy

*C. elegans* hermaphrodites were grown overnight in the dark at 20 °C on NGM plates containing 5 μM TMRE. Hermaphrodites were dissected and embryos were released from the gonads. Embryos were visualized using an Olympus IX83 microscope (Olympus Corp.), which is equipped with a 150 × , 1.45 N.A. objective, an EM CCD camera (Andor iXon+ DU-897D-C00-#BV-500), and the 405, 488 and 561 nm lines of a Sapphire CW CDRH USB Laser System attached to a spinning disk confocal scan head (Yokogawa CSU-X1 Spinning Disk Unit).

### Generation of the *wah-1::gfp* knock-in

The *wah-1::gfp* knock-in was generated using the CRISPR/Cas9 gene editing method^53^,^54^. An injection mix containing 100 ng μl^−1^ *wah-1::gfp* donor plasmid, 20 ng μl^−1^ Cas9 expression construct pDD162 (ref. 53), 40 ng μl^−1^ *wah-1* sgRNA targeting a region very close to the stop codon of the *wah-1* coding region, and 2.5 ng μl^−1^ co-injection markers P_*myo*-2_cherry was injected into young N2 adults. First generation (F1) transgenic animals were cloned out and screened for candidates that contained the desired GFP insertion by PCR. F2 animals homozygous for the GFP knock-in were then isolated and confirmed by DNA sequencing.

### PCR detection of *uaDf5* mtDNA

MTR-stained males were mated with unstained hermaphrodites. Hermaphrodites with strong MTR-labelled spermathecae (full of MTR-stained sperm) were selected and dissected to obtain cross-fertilized embryos. Different stages of cross-fertilized embryos were screened using the Nomarski optics, selected and subjected to two rounds of nested PCR analysis to detect the *uaDf5* paternal mtDNA deletion. To obtain cross-fertilized larvae and adults, cross-fertilized embryos were rescued from the agar pads, transferred to normal NGM plates, and allowed to hatch out and grow to the desired developmental stages before they were subjected to the PCR analysis to detect the *uaDf5* deletion. Primers for the first round PCR: P1 5′ GATTAGCACAAGCTTTATTGGATGG 3′ and P2 5′ AAGATCTTAACATTCCGGCTGAGGC 3′^9^,^10^,^11^. The second round primers are: U1-F 5′ CCATCCGTGCTAGAAGACAA 3′ and Cemt1A-R 5′ CTTCTACAGTGCATTGACCTAGTC 3′^10^. Worm lysates were prepared from eight embryos or animals and used as templates for the nested PCR analysis.

### Electron microscopy and ET imaging

Mated *C. elegans* hermaphrodites were mixed with *E. coli* slurry and the mixture was rapidly frozen with an HPM 100 high-pressure freezer (Leica Microsystems, USA). Frozen samples were freeze-substituted in anhydrous acetone containing 2.0% osmium tetroxide for 48 h in −80 °C. The temperature was slowly increased to −20 °C over 24 h and to 4 °C over 8 h. After incubating for 30 min at room temperature, the samples were rinsed three times with anhydrous acetone and embedded in Epon-Araldite resin (Ted Pella, USA) by a stepwise increase in resin concentrations from 0, 25, 50, 75 to 100% over two days. Epoxy resin was polymerized by incubating in 65 °C for 48 h and individual hermaphrodites in the polymerized resin were mounted for preparing serial sections. For conventional TEM imaging, thin sections (80 nm) were collected on copper slot grids (2 mm × 1 mm, Electron Microscopy Sciences, USA) and post-stained with 2% uranyl acetate and Reynold's lead citrate. The sections were examined with a Hitachi 7400 TEM operated at 80 KV (Hitachi High-Technologies, Japan). For tomography analyses, 300 nm thick sections were collected on copper slot grids (2 mm × 1 mm, Electron Microscopy Sciences, USA) and post stained with 2% uranyl acetate and Reynold s lead citrate. After depositing fiducial gold particles (15 nm), tilt series were obtained from +/− 60° at 1.5° intervals around two orthogonal axes with an F20 field emission gun transmission electron microscope operated at 200 KV (FEI, USA). Calculation of dual axis tomogram calculation and modeling subcellular organelle were performed with the IMOD software package (www.bio3d.colorado.edu/imod). Briefly, the tilt series were aligned using the fiducial gold particles as a guide and tomogram positions were determined in sample tomograms. The fully aligned tilt series were converted into tomograms with a back projection algorithm and tomograms from two orthogonal axes were combined by correlating patches between the two orthogonal tomograms. To join tomograms from serial sections, we used the MIDAS interface for alignment and joined the aligned tomograms with the makejoincom and finishjoin programs included in the IMOD package. Toyooka and Kang^55^ have detailed explanation of generating tomograms from serial sections using the etomo interface. Image slices were captured from the 3dmod interface and rendered into movies using the QuickTime Player Pro software (ver. 7.1, Apple, USA).

### Data availability

The data that support the findings of this study are available from the authors upon request.

## Additional information

**How to cite this article:** Wang Y. *et al*. Kinetics and specificity of paternal mitochondrial elimination in *Caenorhabditis elegans*. *Nat. Commun.* 7:12569 doi: 10.1038/ncomms12569 (2016).

## Supplementary Material

Supplementary Figures Supplementary Figures 1-7

Supplementary Movie 1 A movie of electron tomographic slices showing a spermatozoon (right) right next to a fertilized oocyte immediately after sperm entry (left). Paternal mitochondria in the spermatozoon and in the fertilized oocyte are indicated with green and yellow arrowheads, respectively. MO: membranous organelles.

Supplementary Movie 2 A movie of electron tomographic slices of the mitochondria shown in Fig. 4k,l. Three paternal mitochondria (orange arrows) and three fzo-1(tm1133) maternal mitochondria (magenta arrowheads) are seen in the movie. Two of the fzo-1(tm1133) maternal mitochondria were compromised and had a large dark inclusion in their matrix (top left and bottom middle).
