# Supplementary material for: Kinetics and specificity of paternal mitochondrial elimination in Caenorhabditis elegans
Source: Nat Commun. 2016 Sep 1;7:12569. doi: 10.1038/ncomms12569 (PMC5025750; doi:10.1038/ncomms12569)
Supplement: Supplementary Figures — 1-7 [file ncomms12569-s1.pdf]

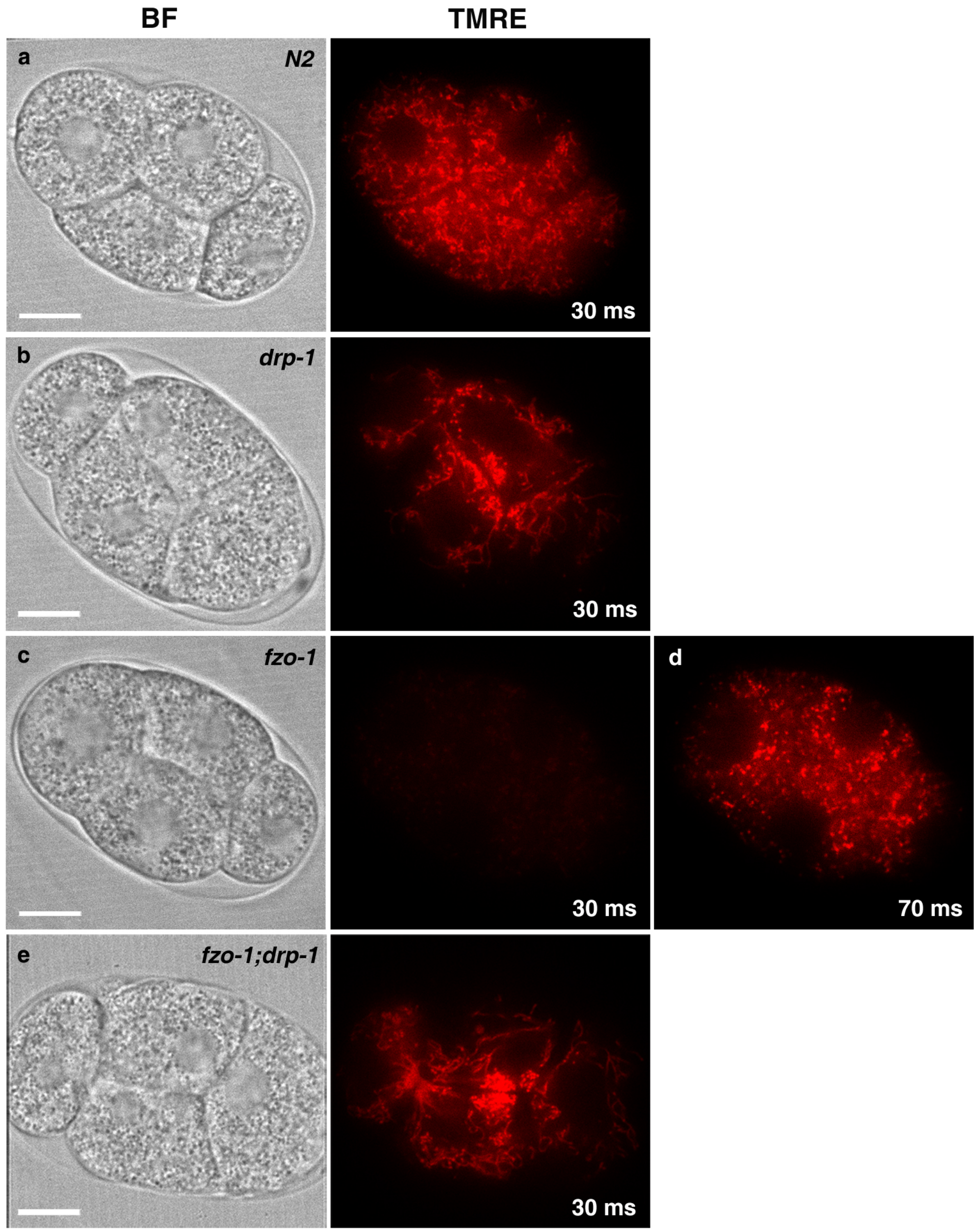

Supplementary Figure 1 Maternal mitochondrial morphology in 4-cell stage embryos from different mutants. Bright-field (BF) and fluorescence images of four-cell stage embryos stained by tetramethylrhodamine ethyl ester (TMRE) are shown. Embryos examined are: wild-type (**a**), *drp-1(tm1108)* (**b**), *fzo-1(tm1133)* (**c,d**), and *fzo-1(tm1133); drp-1(tm1108)* (**e**). The exposure times for the 561 nm laser are 30 ms (**a-c, e**) and 70 ms (**d**), respectively. Scale bar represents 10 μm.

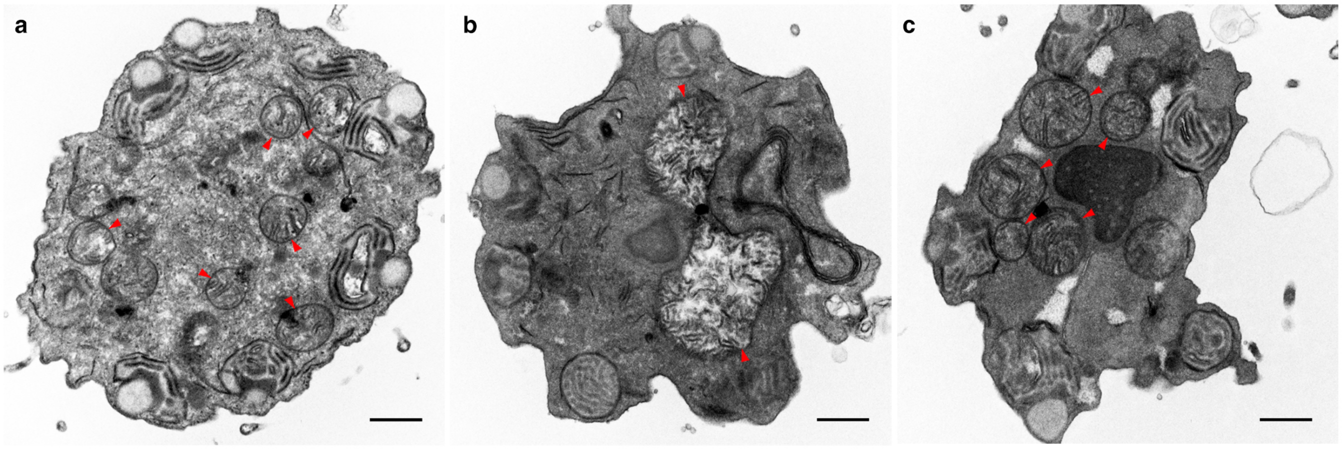

d

genotype	length (μm)	SEM	range	n
N2	0.513	0.010	0.339-0.923	102
drp-1(tm1108)	1.046	0.039	0.526-2.874	107
fzo-1(tm1133)	0.625	0.017	0.284-1.259	109

Supplementary Figure 2 Morphology and sizes of paternal mitochondria in spermatozoa of N2, *drp-1(tm1108)* and *fzo-1(tm1133)* males. Representative electron micrographs of spermatozoa from *N2* (**a**), *drp-1(tm1108)* (**b**) and *fzo-1(tm1133)* (**c**) males. Scale bars represent 0.5 µm. Arrowheads indicate paternal mitochondria. (**d**) Quantification of the mean mitochondrial length. Randomly selected mitochondria from electron micrographs were measured along their longitudinal axis. n, the number of mitochondria measured.

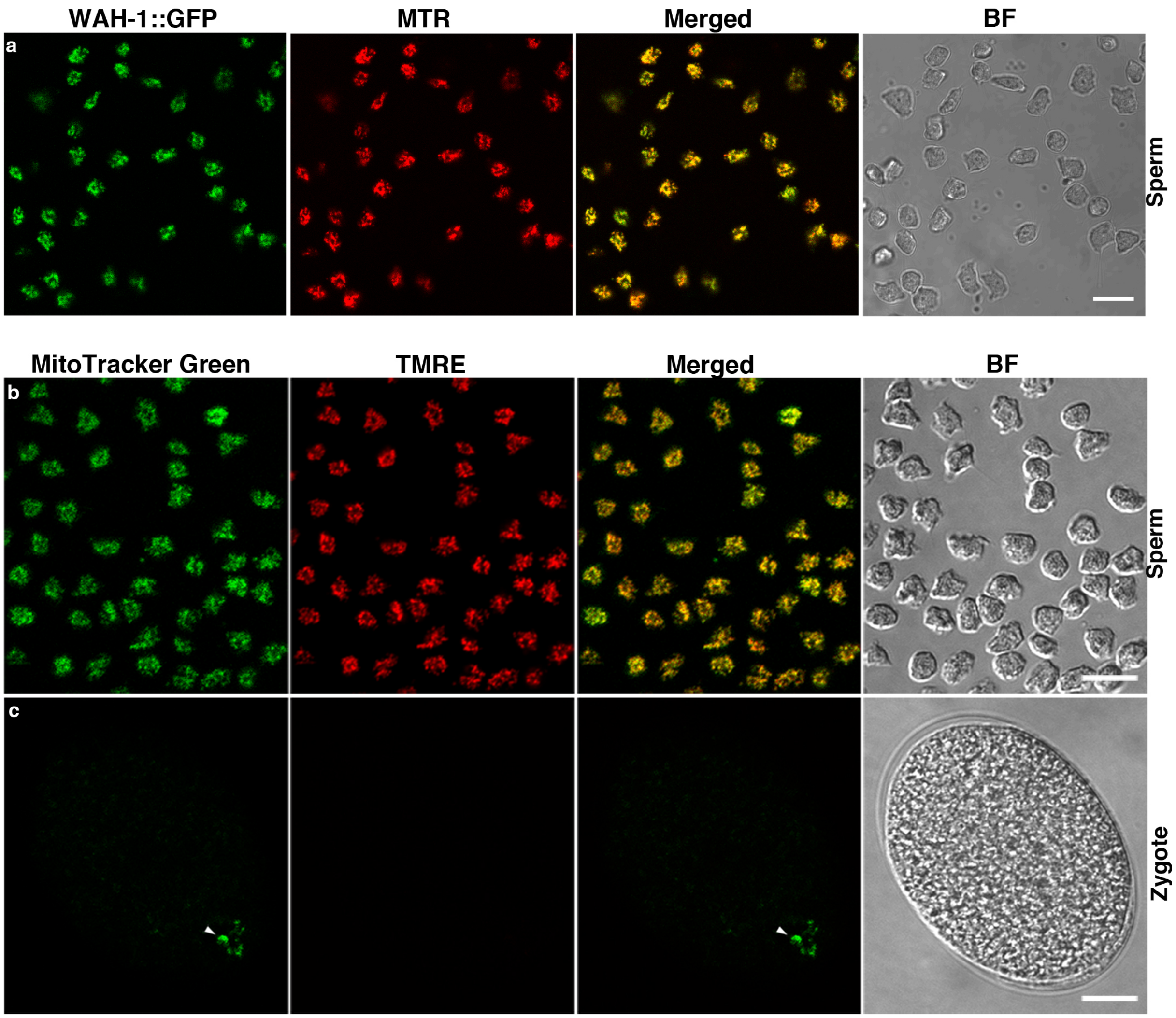

Supplementary Figure 3 Mitochondrial dye staining of paternal mitochondria in spermatozoa and in zygotes. (**a**) MitoTracker Red (MTR) staining of paternal mitochondria in spermatozoa of N2 males carrying the *wah-1::gfp* knock-in allele (see Methods). Confocal images of WAH-1::GFP, MTR, WAH-1::GFP/MTR merged, and the corresponding bright-field (BF) image from spermatozoa released from the gonads of males prestained with MTR are shown. (**b,c**) Confocal images of MitoTracker Green, TMRE, MitoTracker Green/TMRE merged, and the corresponding BF image from spermatozoa released from the gonad of a wild-type male prestained with TMRE and MitoTracker Green (**b**) or from a zygote right after sperm entry from mating of N2 males prestained by TMRE and MitoTracker Green with unstained N2 hermaphrodites. The arrowhead indicates paternal mitochondria labeled by MitoTracker Green. Scale bars represent 10 μm.

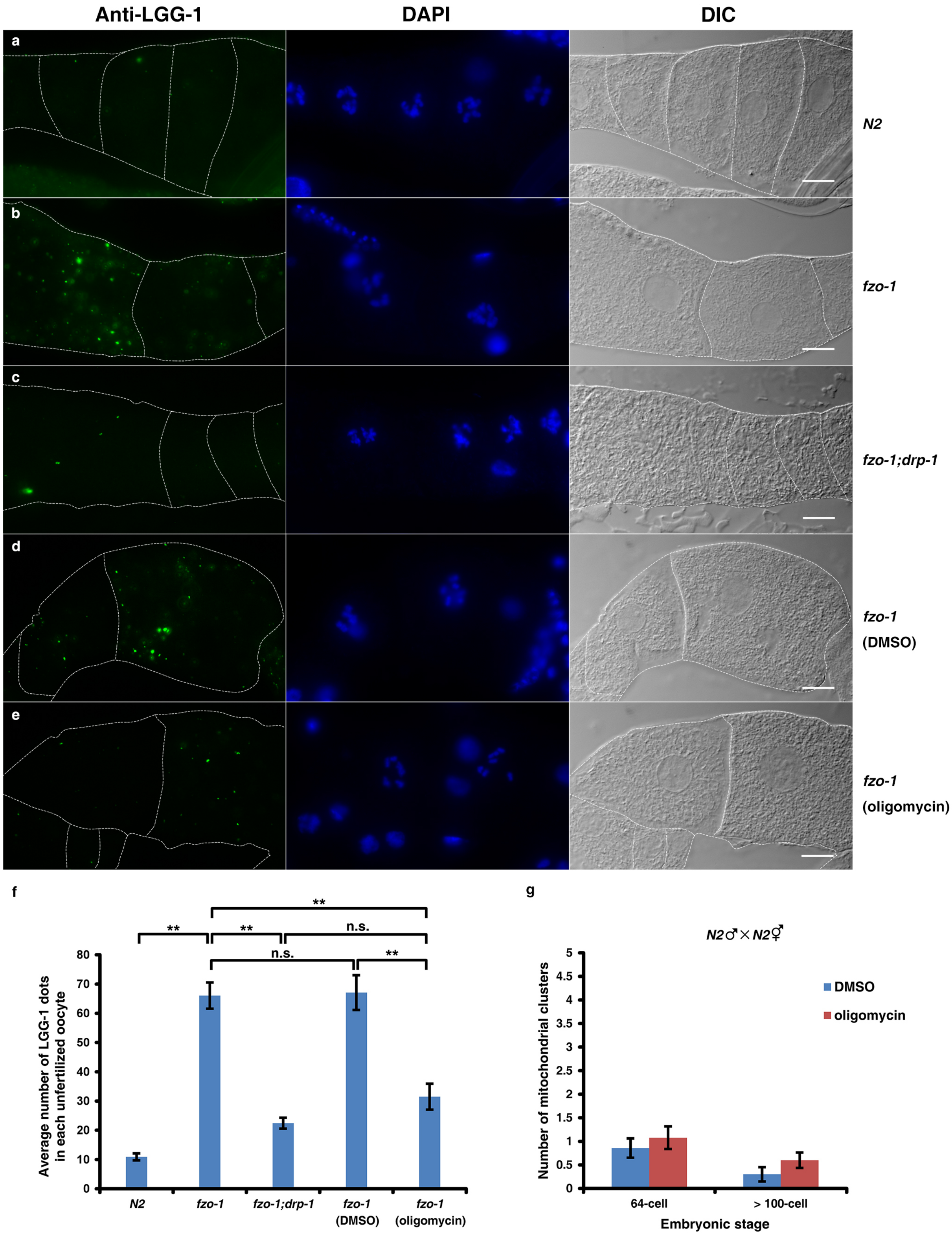

Supplementary Figure 4 Distribution and patterns of LGG-1 staining in unfertilized oocytes of various genotypes**.** (**a**-**e**) Gonads were dissected from hermaphrodites with the indicated genotype grown on regular NGM plates (**a**-**c**), or plates with the DMSO control (**d**) or 150 μg mL^{-1} oligomycin

(**e**), and stained with LGG-1 antibody (green) and DAPI (blue). White dashed lines outline unfertilized oocytes in the gonads. Scale bar represents 10 μm. (**f**) Quantification of the mean numbers of LGG-1 dots in unfertilized oocytes of various genotypes (**a**-**e**). Data shown are mean $\pm$ s.e.m. (n=20 unfertilized oocytes). (**g**) Oligomycin treatment has no effect on PME in cross-fertilized embryos derived from mating of wild type parents. Quantification of MTR-stained paternal mitochondrial clusters in 64-cell or > 100-cell stage embryos from animals grown on NGM plates containing the DMSO control or 150 μg mL^{-1} oligomycin was performed as in **Fig. 2h**. MTR-stained N2 males were mated with unstained N2 hermaphrodites. Data shown are mean $\pm$ s.e.m. (n=10). The significance of difference between different strains or different treatments was determined by unpaired *t* test (**f, g**). ** $P < 0.01$. "n.s." indicates no significant difference. In all panels, *fzo-1(tm1133)* and *drp-1(tm1108)* alleles were used.

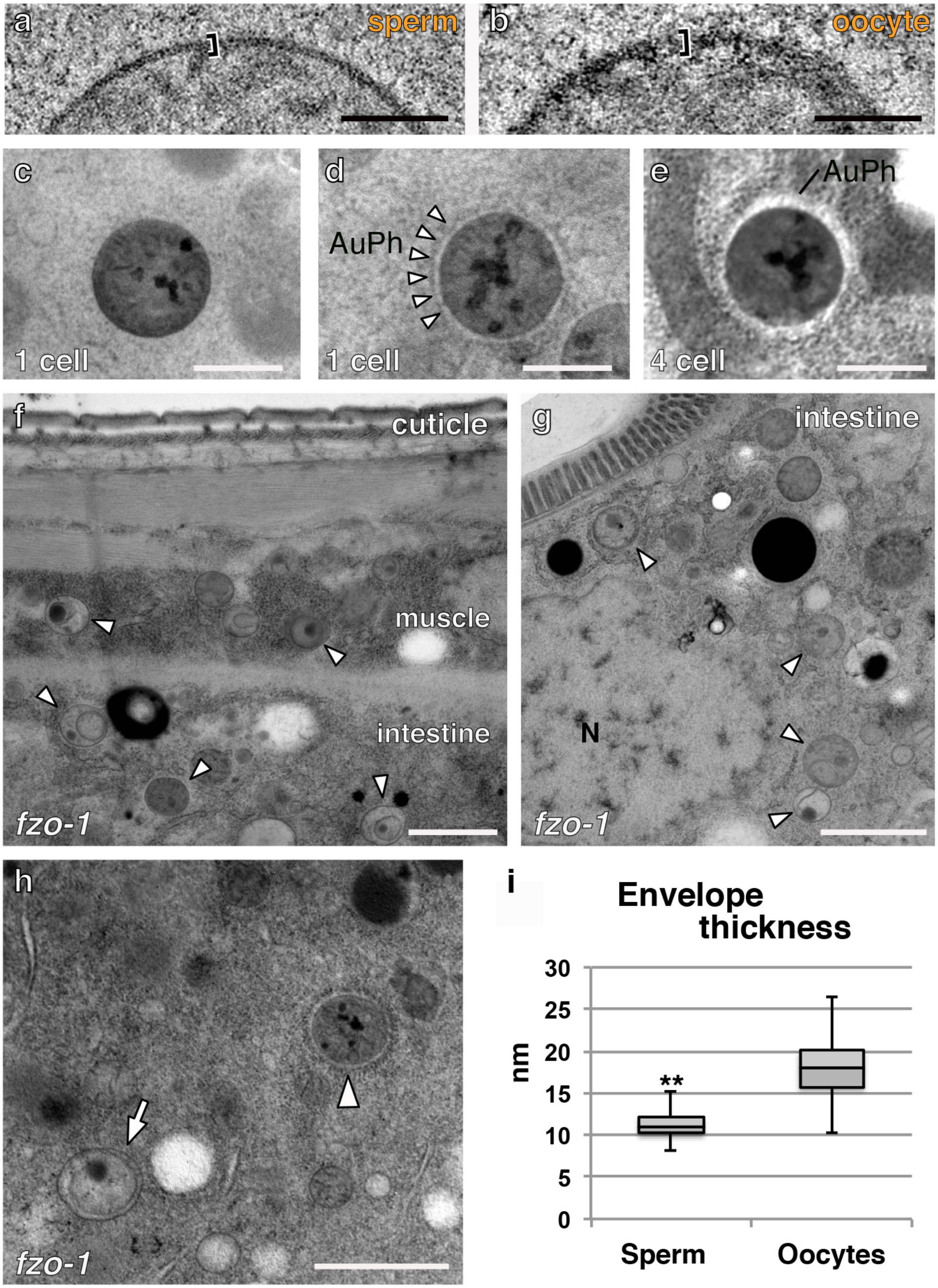

Supplementary Figure 5 Transmission electron micrographs (TEM) and tomographic slice images of mitochondria from N2 spermatozoa and embryos and *fzo-1(tm1133)* somatic cells and embryos. (**a,b)**

Tomographic slice images of sperm mitochondria before (**a**) and after (**b**) fertilization. Note that the sperm mitochondrial membranes in the zygote are blurry and thick (brackets in **a** and **b**). (**c-e**) Different stages of autophagosome enclosure of paternal mitochondria after fertilization. Dark speckles appeared in the matrix of the sperm mitochondrion soon after fertilization, but no autophagosome membrane was observed in its vicinity (**c**). The speckles enlarged as the matrix started to clear and the autophagosome membranes (AuPh) surrounded the sperm mitochondrion (**d**). In the 4-cell stage embryo, the sperm mitochondrion was mostly emptied inside the autophagosome (**e**). (**f**,**g**) Electron micrographs of a muscle cell (**f**) and intestine cells (**g**) in *fzo-1(tm1133)* animals show compromised mitochondria, which are round and have a dark inclusion in their matrix (arrowheads). (**h**) An electron micrograph of a *fzo-1(tm1133)* zygote, in which a paternal mitochondrion was enclosed by an autophagosome (indicated by an arrowhead), whereas a compromised maternal mitochondrion nearby was not enclosed by an autophagosome (indicated by an arrow). Thus the autophagy machinery does not preferentially target maternal or paternal mitochondria in *fzo-1(tm1133)* eggs (see also **Fig. 4m,n**). (**i**) Histogram of mitochondrial membrane thickness. Tomographic slice images of paternal mitochondria in sperm (n=25) and in zygotes (n=23) were imported into ImageJ (imagej.nih.gov/ij/) and the membrane thickness was measured. The significance of difference between two sets of results is determined by unpaired t test. ** $P < 0.0001$. Scale bar in **a,b**: 500 nm. Scale bars in **f,g,h**: 1 μm. Scale bars in **c-e**: 250 nm.

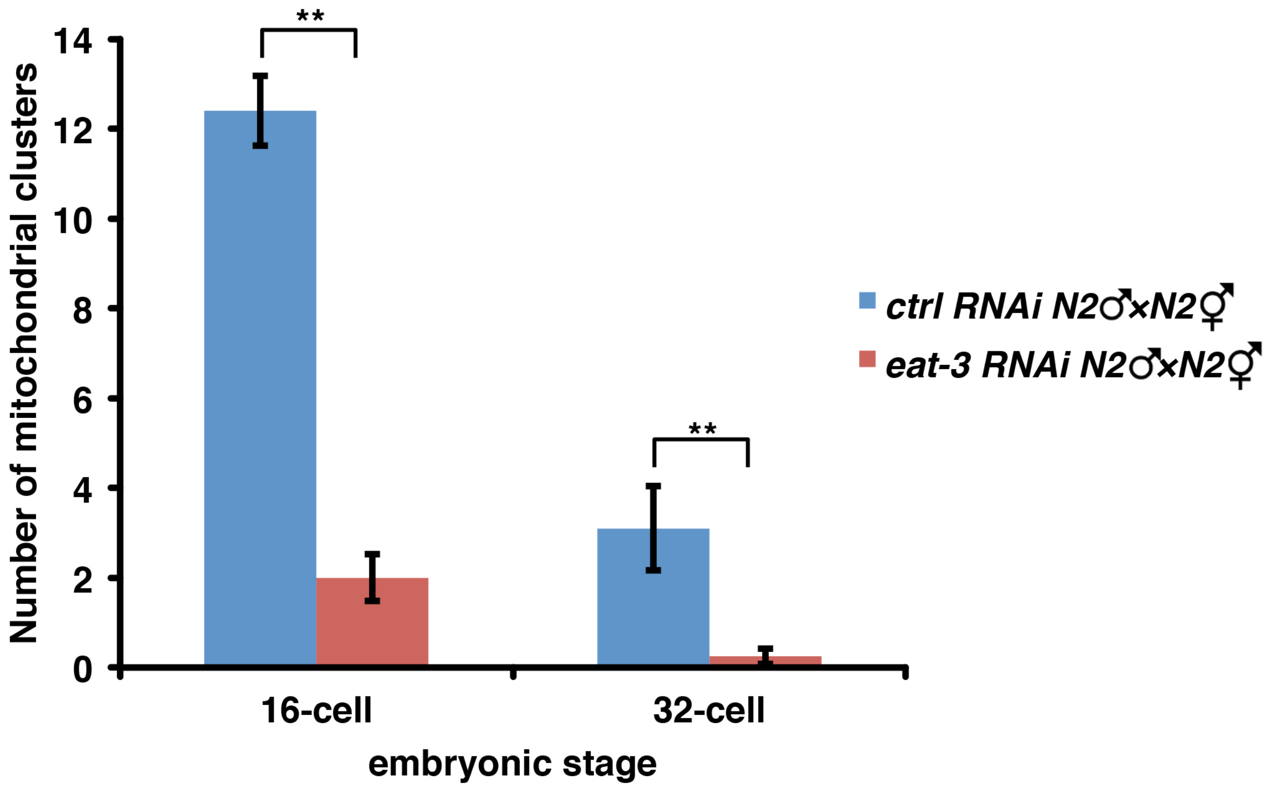

Supplementary Figure 6 *eat-3* RNAi treatment of males accelerates PME. Quantification of MTR-stained paternal mitochondrial clusters in 16- and 32-cell stage cross-fertilized embryos from the indicated crosses was performed as in **Fig. 1h**. L4 stage N2 hermaphrodites were mated with L4 males that were placed on *eat-3* or control RNAi plates for two generations and prestained with MTR on normal OP50 plates overnight. Data shown are mean ± s.e.m. (n=15). The significance of difference between different mating experiments was determined by unpaired t test. ** $P < 0.01$.

Fig. 2i

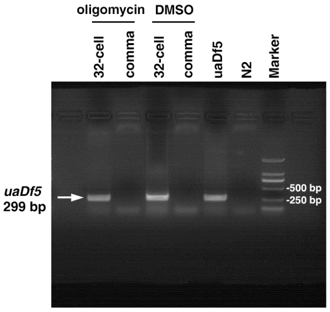

cross progeny from *uaDf5/+*♂×*N2*⚥

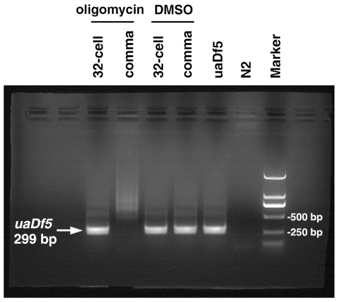

cross progeny from *uaDf5/+*♂×*fzo-1*⚥

Fig. 7b

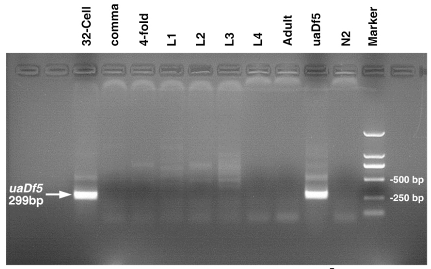

cross progeny from *uaDf5/+*♂×*N2*⚥

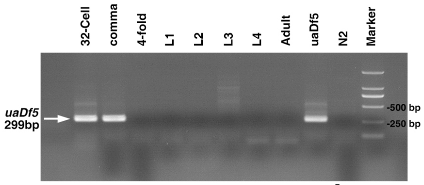

cross progeny from *uaDf5/+*♂×*fzo-1*⚥

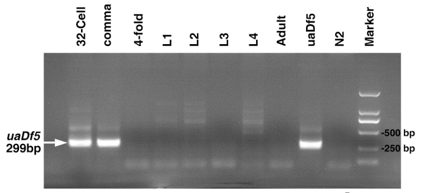

cross progeny from *drp-1;uaDf5/+*♂×*N2*⚥

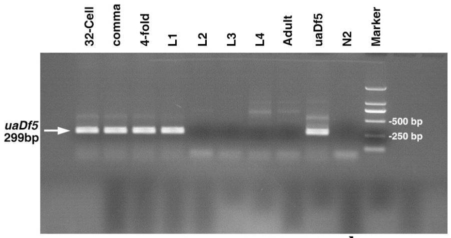

cross progeny from *drp-1;uaDf5/+*♂×*fzo-1*⚥

Fig. 7e

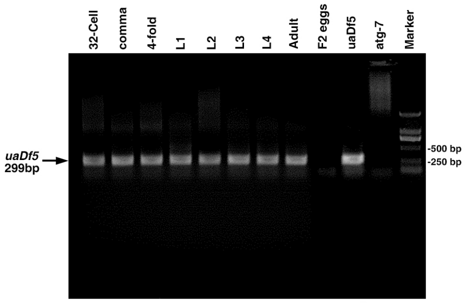

cross progeny from *atg-7;uaDf5/+*♂×*atg-7*⚥

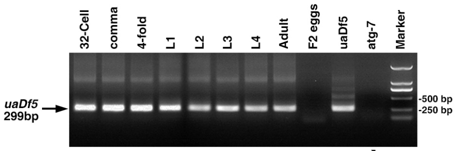

cross progeny from *atg-7;uaDf5/+*♂×*fzo-1;atg-7*⚥

Supplementary Figure 7 Uncropped gel images of **Fig. 2i, Fig. 7b** and **Fig. 7e**.
